# Robust single-shot 3D fluorescence imaging in scattering media with a simulator-trained neural network

**Published:** 2023-03-22

**Authors:** Jeffrey Alido, Joseph Greene, Yujia Xue, Guorong Hu, Yunzhe Li, Kevin J. Monk, Brett T. DeBenedicts, Ian G. Davison, Lei Tian

**Affiliations:** 1 Department of Electrical and Computer Engineering, Boston University, Boston, MA, 02215, USA.; 2 Department of Biology, Boston University, MA 02215, USA; 3 Department of Biomedical Engineering, Boston University, Boston, MA, 02215, USA.

## Abstract

Imaging through scattering is a pervasive and difficult problem in many biological applications. The high background and the exponentially attenuated target signals due to scattering fundamentally limits the imaging depth of fluorescence microscopy. Light-field systems are favorable for high-speed volumetric imaging, but the 2D-to-3D reconstruction is fundamentally ill-posed and scattering exacerbates the condition of the inverse problem. Here, we develop a scattering simulator that models low-contrast target signals buried in heterogeneous strong background. We then train a deep neural network solely on synthetic data to descatter and reconstruct a 3D volume from a single-shot light-field measurement with low signal-to-background ratio (SBR). We apply this network to our previously developed Computational Miniature Mesoscope and demonstrate the robustness of our deep learning algorithm on a 75 μm thick fixed mouse brain section and on bulk scattering phantoms with different scattering conditions. The network can robustly reconstruct emitters in 3D with a 2D measurement of SBR as low as 1.05 and as deep as a scattering length. We analyze fundamental tradeoffs based on network design factors and out-of-distribution data that affect the deep learning model’s generalizability to real experimental data. Broadly, we believe that our simulator-based deep learning approach can be applied to a wide range of imaging through scattering techniques where experimental paired training data is lacking.

Fluorescence imaging is crucial in biological research because it permits observation of gene expression and molecular interactions in cells and tissues^1–4^. One-photon imaging systems, such as widefield microscopes and miniscopes^5–10^, are often employed to visualize and measure fluorescence, but the imaging depth is limited by tissue scattering which degrades and obscures target signals^11–13^. This, along with non-specific fluorescence that contributes to a high level of hazy, spatially heterogeneous background, resulting in measurements with a low signal-to-background ratio (SBR), where objects embedded in deeper layers are nearly imperceptible to both humans and machines.

Light scattering in one-photon fluorescence imaging systems corrupts measurements in an almost non-invertible way, of which the most significant aspect impairing image formation is the attenuation of ballistic photons and increased background contributions^5,10,14^. A conventional approach to recover signals from scattering-contaminated fluorescence measurements is to perform background removal using standard image processing algorithms^5,15,16^. The drawback of this approach is that low-contrast target signals are often removed along with the background since the algorithm cannot discriminate low-SBR target signals from the background. As a result, it is difficult to reconstruct objects beneath shallow layers of scattering media as their signals carried by the ballistic photons attenuate exponentially and are further obscured by strong background. While this results in measurements with low SBR, the object information is not completely lost^17,18^. The challenge becomes how to robustly utilize the information encoded in the scattered light in the presence of strong background.

Recently, deep learning methods have been applied to imaging through scattering^19–27^ due to its ability to solve highly ill-posed inverse problems. An outstanding challenge to apply deep learning techniques in real experiments is how to collect training data. One strategy is to collect experimental paired data to learn the inverse mapping to descatter images and reconstruct high contrast objects^19,20^; however, obtaining ground-truth reference is costly and time-consuming. Another strategy is to employ a physics-based imaging model to aid in learning the inverse mapping by either integrating it directly in a self-supervised learning scheme^21,22^ or by generating synthetic paired training data offline for supervised learning^23–28^. While some of these methods have shown state-of-the-art results, they are limited to reconstructing objects from measurements under the specific scattering and imaging conditions on which they were trained. Therefore, these methods are less robust to out-of-distribution data caused by experimental deviations that are common in biomedical imaging applications, such as background fluorescence and low SBR.

The requirement of high-speed volumetric imaging is common in biomedical research^2^. Light-field microscopy is a computational imaging technique that enables single-shot 3D fluorescence imaging^5,6,8,10,29–32^. However, the ill-posedness of the 2D-to-3D light-field inverse problem is exacerbated by scattering. Nonetheless, light-field measurements contain useful information within the tomographic redundancies that can aid with imaging through scattering^10,14,31,33,34^. For example, the angular information in a light-field measurement augmented with temporal fluctuation information of the fluorescent objects allow devising physics-based 3D reconstruction algorithms robust to volumetric scattering^14,31^. A recent study developed an incoherent multi-layer model for scattering-robust 3D fluorescence reconstruction without temporal information^35^. However, these methods generally suffer from high model complexity that renders the inversion to be highly time intensive.

Here, we present a robust supervised deep learning approach for single-shot 3D fluorescence imaging through scattering. We demonstrate our method on our previously developed light-field-based Computational Miniature Mesoscope (CM^2^)^5,6^ imaging platform (Fig. 1a, see Methods for details). We show in experiment that our method provides comparable depth penetration and sectioning capability to that of one-photon confocal microscopy in highly scattering media. To overcome the challenge of generating paired training data, we devise a synthetic data generation process that integrates the scattering-free imaging model of CM^2^ and a synthetic scattering-induced background model. Importantly, the synthetic background model is computationally simple and highly generalizable to different scattering conditions. The added background contains random slow variations to account for inhomogeneities due to tissue scattering that are modeled by low-pass filtered value noise^36^, and the background strengths are varied to model different SBR levels (Fig. 1b).

To utilize the angular information contained in the light-field measurement for 3D reconstructions, we adapted our previously established network design that combine the functionalities of view-synthesis and enhancement of light-field refocusing with a dual-branch structure^6^ (Fig. 1c). Furthermore, we improve the network, termed SBR-Net, by a new variance stabilization strategy to improve learning convergence in Res-Net style architectures and the information fusion of the two branches, which we demonstrate a 7x speed up in training convergence. Details on the network architecture and the training details are provided in Methods and Supplementary Information.

Our training process of SBR-Net is designed to generalize to different scattering conditions. We generate paired training data by simulating diverse and low SBR light-field measurements with SBRs ranging from 1.1 to 3.0, and their corresponding ground-truth volumes (see Methods for details). The network takes input of the low-SBR data and reconstructs fluorescent emitters in 3D embedded in scattering media. Our simulation results and quantitative analysis show that SBR-Net can accurately reconstruct emitters located at depths up to one scattering length deep inside the scattering media across a broad range of SBR and scattering conditions. To compare with more traditional inverse scattering reconstruction approaches, we also train a network on free space data, termed FS-Net. The input of FS-Net is the scattering measurement processed with a traditional background removal algorithm, and we find poor generalizability and severely worse performance compared to SBR-Net. The spatially varying background signal from our synthetic background model, when added to the free space measurement, mimics the loss of contrast from light scattering as well as from unwanted background signal from non-specific fluorescence. This provides SBR-Net with generalizability across different scattering conditions.

A major achievement we demonstrate is that SBR-Net trained solely on synthetic data generalizes well to real challenging experiments. Using CM^2^ as testbed paired with the simulator-trained SBR-Net, we demonstrate single-shot 3D reconstruction from a 2D measurement of fluorescent objects where their signals have SBRs as low as 1.05. We demonstrate our algorithm in real experiments on a fixed mouse brain slice and on three controlled scattering phantoms of scattering lengths of 279, 182, and 72 μm with added fluorescent background where the target object is a mixture of 10 and 15 μm fluorescent beads. We benchmark the results with confocal microscopy measurements. SBR-Net recovers emitters embedded as deep as one scattering length in all scattering phantoms with 25 μm axial resolution and generalizes to the complex biological structure of brain tissues.

In addition, we provide insights into how to design robust deep learning models for scattering inverse problems. Our detection-based analysis of our algorithm reveals how the SBR range in simulated training data affects the generalization to real experimental measurements, where the choice of SBR range in the training data has a tradeoff between the imaging depth penetration and false positives, equivalently, the robustness-accuracy tradeoff. We also analyze the bias-variance tradeoff based on the network architecture and the number of unique training data pairs to understand the considerations for network generalization performance. Broadly, we believe that our simulator-based deep learning approach for improving reconstruction from low contrast measurements may open up avenues to improve computational strategies for imaging through scattering in many applications, such as neural imaging and intravital imaging.

## Results

### Statistical comparison of simulated and experimental measurements

**Extended Data Fig. 1 |:**
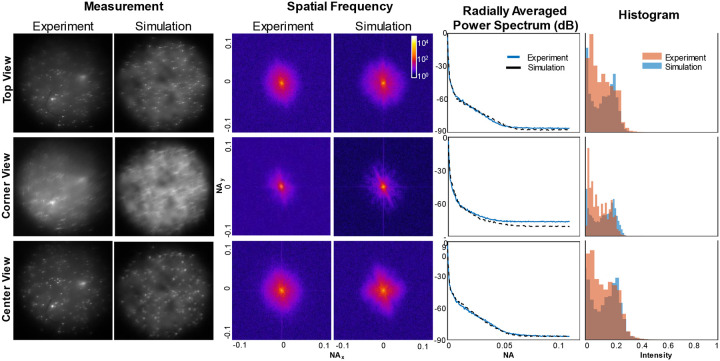
Qualitative and quantitative comparison of synthetic and experimental data for *l*_*s*_ = 279 μm. We show the spatial frequency, power spectra, and intensity histograms for different views of a CM^2^ measurement and observe strong similarities.

We first begin by comparing the statistical properties of experimental and synthetic data to validate our scattering and background model. As shown in Extended Data Fig. 1, and Figs. S2 and S3, the synthetic measurement shares visual qualities with experimental measurements. Additionally, our background model provides good statistical matching with experiment, given by comparing their spatial frequencies, power spectra, and intensity histogram, which is shown to be an important reason for the robustness of neural networks^37^. We also perform principal component analysis (PCA) for each measurement domain, shown in Extended Data Fig. 7a, and see that the first two principal components of the simulated scattering measurement clusters closely with those of experimental scattering measurements.

### Simulated fluorescent beads reconstruction

We first quantify the performance of the trained SBR-Net on synthetic scattering samples under different scattering conditions. Different from the training data, we further incorporate a signal attenuation model in the testing data to assess the performance of SBR-Net on more realistic and quantifiable scattering scenarios. We model this attenuation according to Beer-Lambert’s law, neglecting absorption, given by I(*z*) = I_0_/exp(−*z/l*_*s*_) where *l*_*s*_ is the scattering mean free path / scattering length (see more details in Methods). We experimented on three different synthetic phantoms with scattering lengths of 80, 160, and 320 μm.

**Fig. 2 |:**
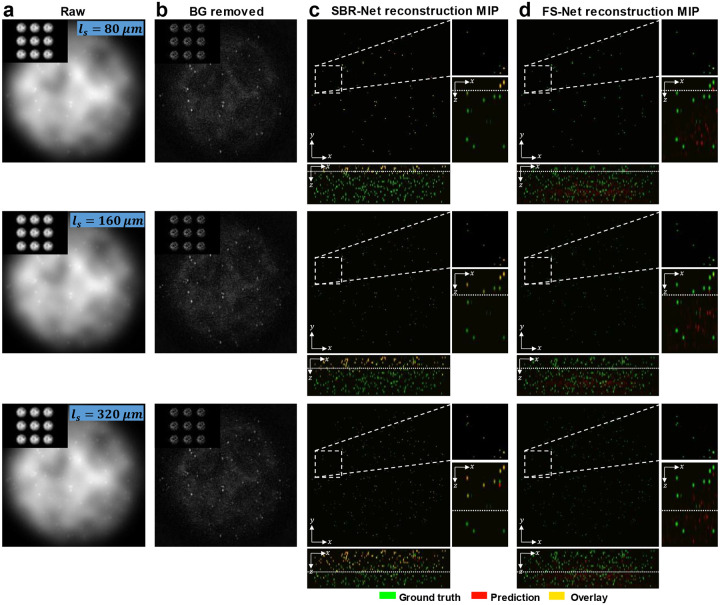
Qualitative reconstruction results for synthetic scattering phantoms. The scattering lengths of the test samples grow quadratically, including Row 1: *l*_*s*_ = 80μm, Row 2: *l*_*s*_ = 160μm, and Row 3: *l*_*s*_ = 320μm. (a) Raw measurement of the central view with the full 3×3-view measurement as the inset. (b) Background (BG) removed measurement. (c,d) Maximum intensity projections (MIPs) of the reconstructions of (c) SBR-Net and (d) FS-Net. The ground-truth particles are shown in green, the predictions in red, and the overlay in yellow. XY MIPs are shown for volumes as deep as one scattering length. The dotted line in the XZ MIPs represent a distance of one scattering length, and we visually observe good emitter reconstruction and localization up to one scattering length for SBR-Net, and almost no predicted emitters beyond one scattering length. FS-Net performs poorly even within shallow layers, while also generating false positives.

**Fig. 3 |:**
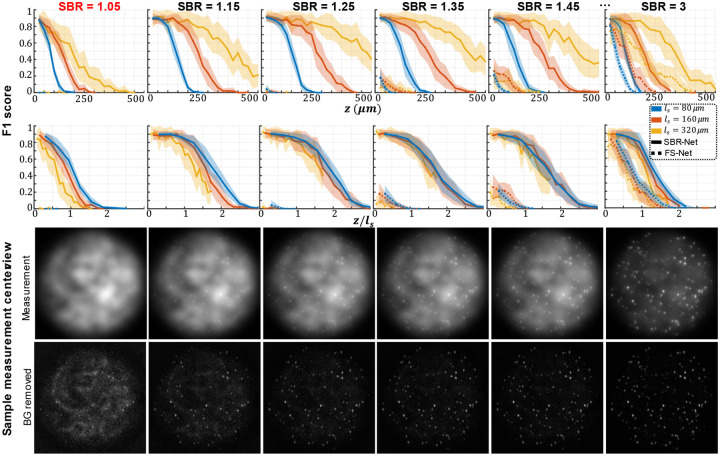
Simulation test data F1 score and visualization of different SBR measurements. The localization performance over physical and normalized depth for different scattering length samples is shown for increasing SBR. Each column corresponds to data with the labeled SBR and the images are sample measurements of data with the labeled SBR. The labeled SBR values are the average SBR values for the emitters in the first depth layer before any optical attenuation. Similar to the optical signal, the SBR of an emitter would decay exponentially over increasing depth of the emitter, which is why we observe a systematic decay of performance for all scattering lengths, as seen in the normalized depths curves. The line represents the average over 25 samples and shaded regions represent standard error.

In Fig. 2, we visually assess the SBR-Net reconstructions, which is further quantified using the F1 score over depths for all SBR from 1.05 to 3.0 in Fig. 3 and Fig. S5. When examining the results with physical depth (*z*), we see that the F1 score generally decays with depth. The larger the scattering length (*l*_*s*_) the slower the decay rate, indicating a deeper imaging capability compared to samples with shorter scattering lengths. To provide additional physical insights, we further examine the curves with the normalized depth by the scattering length (*z/l*_*s*_). For a given SBR, the three curves corresponding to different scattering lengths concentrate around the same trend line. In all SBR cases, SBR-Net is able to reconstruct emitters in 3D with an F1 score of around 0.9 for up to one scattering length. Higher SBR generally results in slightly improved performance at deeper depths. SBR-Net was trained on scattering measurements with an SBR range of 1.1 to 3.0, which explains why there is weaker depth penetration for the out-of-distribution SBR = 1.05 case. However, we show in our ablation study that it is undesirable to train SBR-Net on lower SBRs due to poorer generalization performance to experimental data. The results for the SBR = 3.0 case are slightly worse than those of lower SBR, which can be explained by the network having seen more examples of images with lower contrast than higher, resulting in poorer reconstructions for higher contrast data.

To benchmark the performance, we also trained FS-Net on background removed scattering measurements (see Methods). SBR-Net significantly outperforms FS-Net in terms of reconstruction accuracy and depth penetration, where SBR-Net has a higher F1 score over all depths for all SBR from 1.05 to 3.0. Additionally, SBR-Net has a higher precision and recall score than FS-Net, shown in Fig. S5. FS-Net performs poorly due to the background removal step removing target emitters along with the background. Emitters embedded more deeply in scattering tissue are attenuated more strongly according to Beer-Lambert’s law and thus have a lower SBR, making its morphological features similar to that of the slowly varying background signal. Additionally, there are severe hallucination artifacts.

### Experimental scattering phantoms reconstruction

**Extended Data Fig. 2 |:**
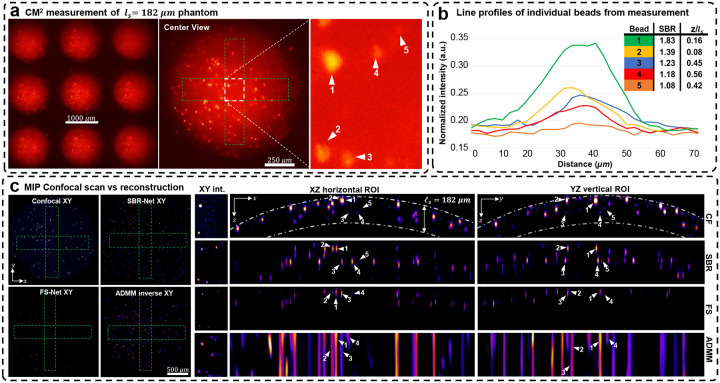
Reconstruction results for the *l*_*s*_ = 182 μm phantom scattering phantom. (a) The raw CM^2^ measurement with a zoom-in of a small region of interest (ROI) with beads we label to validate reconstruction performance. (b) Line profiles of the labeled beads in the raw CM^2^ measurement. We list each bead’s depth relative to the surface of the phantom as well as their measurement SBRs. (c) MIPs of confocal microscopy 3D measurements, reconstructions with SBR-Net, FS-Net, and the model-based ADMM algorithm. The dashed-dotted line in the confocal XZ/YZ MIPs represent a distance of one scattering length from the surface of the phantom. For this scattering phantom, SBR-Net is able to recover all 5 beads at their correct depth location, which is as deep as over half a scattering length. SBR-Net also reconstructs and localizes a particle with a measurement SBR of 1.08. However, while SBR-Net localizes particles well, it reconstructs them with some inaccuracies in intensity and size. FS-Net and ADMM both fail to reconstruct the particle of the 5 with the lowest SBR of 1.08 and localizes the remaining 4 with poor accuracy. Additionally, the XY MIPs show that SBR-Net provides background rejection for the 2D reconstruction case while retaining low SBR particles, which FS-Net and ADMM remove.

**Extended Data Figure 3 |:**
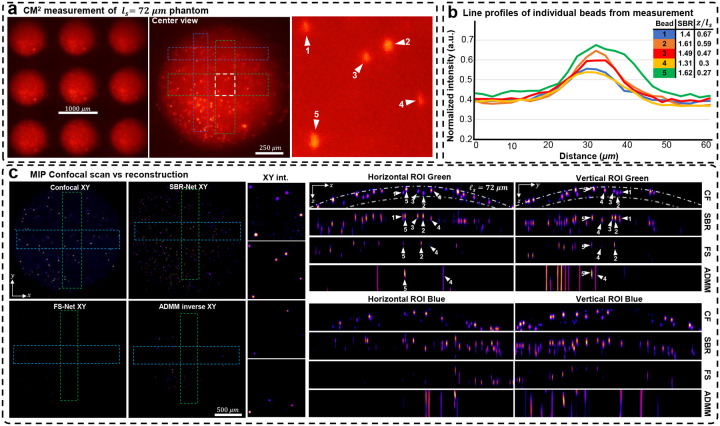
Reconstruction results for the *l*_*s*_ = 72 μm phantom. (a) The raw CM^2^ measurement with a zoom-in of a small region of interest (ROI) with beads we label to validate reconstruction performance. (b) Line profiles of the labeled beads in the raw CM^2^ measurement. We list each bead’s depth relative to the surface of the phantom as well as their measurement SBRs. (c) MIPs of confocal microscopy 3D measurements, reconstructions with SBR-Net, FS-Net, and the model-based ADMM algorithm. The dashed-dotted line in the confocal XZ/YZ MIPs represent a distance of one scattering length from the surface of the phantom. For this scattering phantom, SBR-Net is able to recover all 5 beads at their correct depth location. FS-Net and ADMM both fail to reconstruct all 5 particles. Across a large FOV, we see consistent performance of SBR-Net reconstructing low SBR particles, while FS-Net and ADMM fail to reconstruct them.

**Fig. 4 |:**
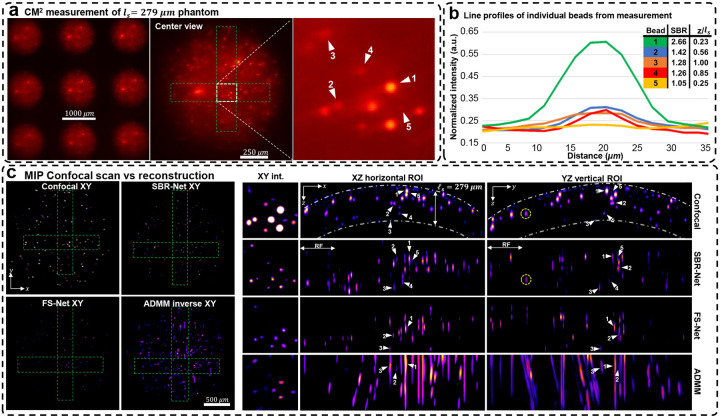
Reconstruction results for the *l*_*s*_ = 279 μm scattering phantom. (a) The raw CM^2^ measurement with a zoom-in of a small region of interest (ROI) with beads we label to quantify reconstruction performance. (b) Line profiles of the labeled beads in the raw CM^2^ measurement. We list each bead’s depth relative to the surface of the phantom (based on confocal measurements) as well as their measurement SBRs. (c) MIPs of confocal microscopy 3D measurements, reconstructions with SBR-Net, FS-Net, and the model-based ADMM algorithm. The dashed-dotted line in the confocal XZ/YZ MIPs represent a distance of one scattering length from the surface of the phantom. For this scattering phantom, SBR-Net can recover all 5 beads at their correct depth location, which is as deep as a complete scattering length. SBR-Net also reconstructs and localizes an emitter with a measurement SBR of 1.05. FS-Net and ADMM fail to reconstruct 2 emitters of the 5 with the lower SBRs and localize the remaining 3 with poor accuracy compared to that of SBR-Net. Additionally, the XY MIPs show that SBR-Net provides background rejection while retaining low SBR emitters, which FS-Net and ADMM remove.

We experimentally evaluate our reconstruction model on three scattering phantoms of different scattering lengths, including 72, 182, and 279 μm. In Fig. 4, we show the raw CM^2^ measurements and quantify emitters’ SBR and depth in a small region of interest (ROI) for the *l*_*s*_ = 279 μm phantom. We also show the XZ and YZ MIPs for ROIs of the reconstruction across the object’s FOV and inspect localization and reconstruction accuracy. We benchmark our deep learning results against confocal microscopy measurements and our previously developed model-based reconstruction algorithm^5^ (see details in Methods). The results for the *l*_*s*_ = 182 and 72 μm phantoms are shown in Extended Data Figs. 2 and 3.

Our experimental results follow similarly to simulation results; SBR-Net can reconstruct and localize emitters up to one scattering length, comparable to the depth penetration and optical sectioning performance of confocal microscopy. SBR-Net reconstructs emitters with SBR as low as 1.05 in experimental measurements of scattering phantoms, and as deep as one complete scattering length, as shown in Fig. 4b. Across all three scattering phantoms, SBR-Net can reconstruct emitters in 3D with higher fidelity as verified by the confocal measurements. Both FS-Net and the model-based algorithm are limited to reconstructing emitters in shallow layers and suffer from low sensitivity to low-SBR emitters likely because they have been removed by the background removal step. In addition, SBR-Net consistently recovers emitters with ~25 μm axial resolution in all scattering phantoms, whereas the model-based algorithm suffers from much worse axial elongation (~200 μm) similar to our previous results^5^.

While SBR-Net localizes emitters well, it may reconstruct them with some inaccuracies in intensity and size. SBR-Net may also fail at having consistent accurate depth localization relative to neighboring emitters. Figure 2c shows an emitter encircled in yellow that is displaced axially relative to the correctly predicted group of emitters to its left and right. This can be explained by the emitter being one receptive field (RF) length (344.45 μm) away from the edge of the volume. In addition, there are also refraction effects from the dome geometry of the phantom (see the XZ view of the confocal microscopy image of the whole sample in Fig. S6), which we do not account for in our forward model. Refraction causes the experimental measurement to have laterally displaced emitter locations compared to our free space model, which is more severe for the non-central microlenses’ view. Consequently, emitters near the edge, positioned beneath a surface with a normal vector that makes a large angle with the optical axis would suffer from more refraction, resulting in poor axial localization in the reconstruction.

### *Ex-vivo* rodent brain slice reconstruction

**Extended Data Fig. 4 |:**
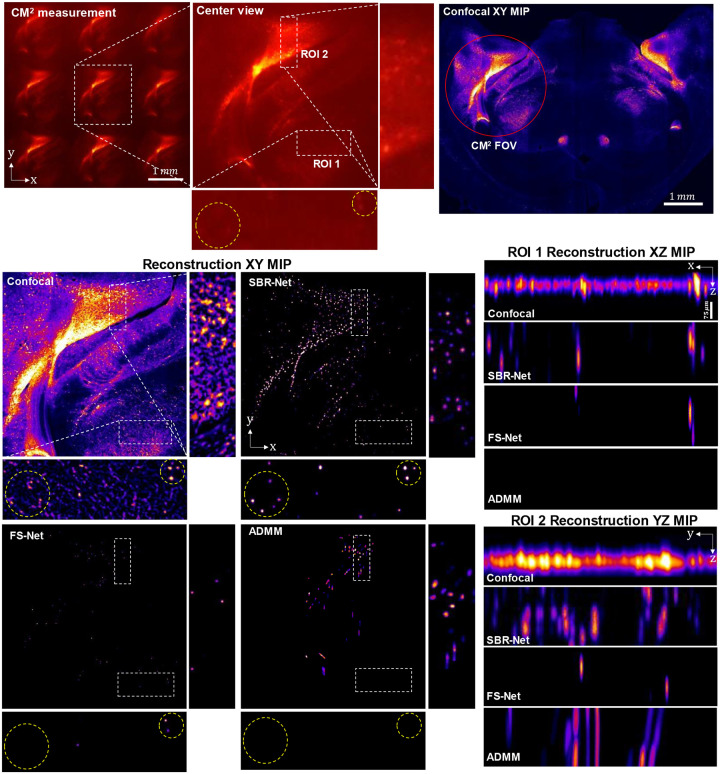
Fixed brain slice reconstruction results with comparisons with FS-Net and model-based inversion. The raw measurement is shown and low SBR cell body clusters from ROI 1 are encircled with a yellow circle. The reconstruction results for SBR-Net, FS-Net, and model-based reconstruction with ADMM is shown with the confocal microscopy measurements as a benchmark. The XZ and YZ MIPs of the ROIs are given to examine the 3D localization accuracy. The XY MIPs reveal that SBR-Net can recover the low SBR signals in ROI 1, while FS-Net and ADMM cannot. SBR-Net has strong localization performance for both the 2D and 3D localization compared to the other methods. FS-Net fails to reconstruct several emitters, but has no false positives, similar to ADMM. Assessing the YZ MIP of ROI 2, SBR-Net trades off 3D localization accuracy for robustness, as it may have incorrect 3D localization, but strong 2D localization, representing accuracy and robustness, respectively.

**Extended Data Fig. 5 |:**
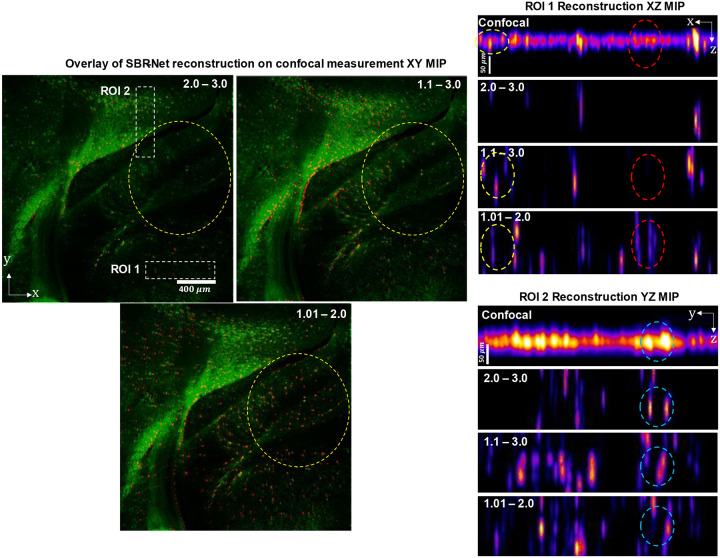
Fixed brain slice reconstruction results for all three SBR-Nets with different SBR range in training. We observe the robustness-accuracy tradeoff in the sparse labelling region of ROI 1 where SBR-Net (1.1 – 3.0) reconstructs fewer emitters compared to SBR-Net (1.01 – 2.0) (red dashed oval), but has more 3D localization accuracy, as highlighted in the yellow dashed oval. While SBR-Net (1.01 – 2.0) may recover more in the 2D reconstruction of the XY MIP, the 3D localization performance is poor, demonstrating a tradeoff of accuracy for robustness. For the denser labelling region of ROI 2, SBR-Net (2.0 – 3.0) performs poorly in robustness, recovering fewer emitters, but has better localization accuracy (light blue dashed oval). As a separate note, SBR-Net (1.01 – 2.0) outperforms the other two networks in XY localization as seen in the XY MIP overlays on confocal measurements.

To demonstrate SBR-Net’s generalization capability on complex biological samples, we applied the simulator-trained SBR-Net directly on a 75-μm thick fixed section of mouse brain containing fluorescently labelled neurons expressing green fluorescent protein (GFP) delivered with virally mediated approaches (see Methods for details). Thickness of the sample is approximately one scattering length of rodent brain tissue^38^. To avoid view-multiplexing in the measurement, we constrain the object FOV to be approximately a circle of 2 mm in diameter by placing a circular field stop directly in front of the sample^5^. The measurement area contains regions with both dense and sparse labelling of neurons. We validated the reconstruction and 3D localization performance of SBR-Net using a confocal microscopy measurement as reference. Comparisons between SBR-Net trained on different SBR ranges (see the next section for details), FS-Net and model-based reconstructions are also provided in Extended Data Figs. 4 and 5.

**Fig. 5 |:**
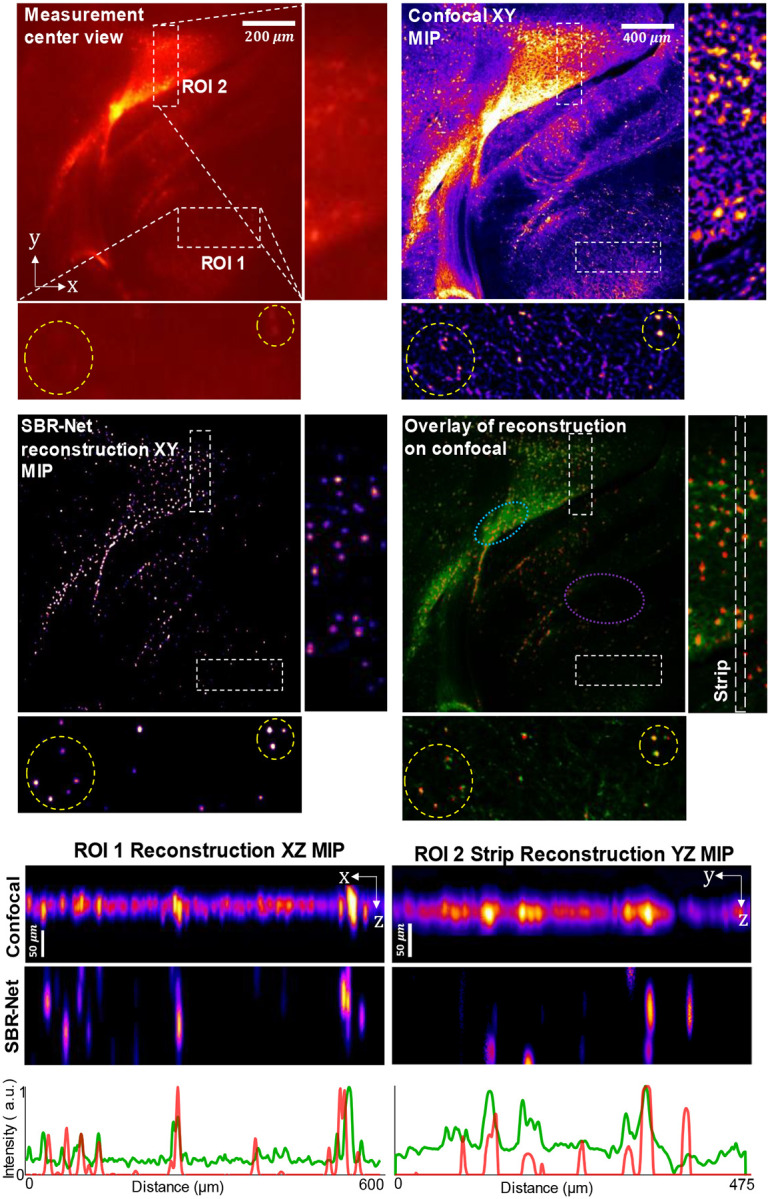
Reconstruction results from an ex-vivo rodent brain slice experiment. ROI 1 is of a region of sparse neuron population, and ROI 2 is of a more densely populated region of neurons. We highlight the reconstruction performance of SBR-Net by marking two clusters of low SBR neurons with a yellow circle in ROI 1 for the measurement and reconstruction MIP. The optical contrast of the encircled neurons is low, with SBR less than 1.2. The confocal XY MIPs of the ROIs are processed with rolling ball background subtraction for better visualization of the neurons. The overlay shows the SBR-Net reconstruction in red, and the confocal measurement in green. The light blue dotted oval shows likely false positive reconstructed neurons from a dense nearly uniform region. The purple dotted oval highlights that SBR-Net recovers only spherical neuronal signals and rejects other structures like dendrites. The line profile is the projection of intensity values along each column, which highlights cell body structures from high background in the confocal measurement MIP.

Our results are shown in Fig. 5, where we demonstrate SBR-Net’s optical sectioning and 3D reconstruction performance with two representative ROIs. ROI 1 is of a denser neuron population with neuropil contamination causing low-SBR measurements. ROI 2 is of a sparse region with dimmer fluorescence that also causes low-SBR measurements. SBR-Net is able to reconstruct neurons from their low-SBR measurements as low as 1.05. In addition, SBR-Net can recover the relative depth position of individual neurons, verified by the confocal microscopy measurement.

Furthermore, SBR-Net rejects some background fluorescent structures like dendrites, recovering only the cell bodies. This can be explained by the network being trained on *only* spherical objects, providing a deep prior that allows the network to reject any other anatomical features. Looking at the overlay between SBR-Net reconstruction and the confocal microscopy measurement, we observe that SBR-Net fails to reconstruct some neurons at the edge of the field of view. This is likely due to the shift-variant aberrations in CM^2^ system^6^ that are exacerbated by scattering leading to distorted features in the measurement that the network has not seen during training, thus it rejects the signals leading to false negatives. We also see that a uniform fluorescent region results in false positive hallucinations. This region is equivalent to a signal with SBR ≈ 1, where the neurons are indistinguishable from the fluorescence background. These hallucinations can be explained by the fact that this SBR ≈ 1 structure is unseen by the network resulting in high variance reconstruction behavior. We analyze this further in a later section.

These results show a promising step towards practical applications of SBR-Net for neural imaging. Recovering signals from their low-SBR measurements may allow improved imaging depth penetration capabilities for miniscope experiments, where neuronal signals embedded deeper in scattering tissue tend to have lower SBRs.

### Effect of training data SBR on network robustness-accuracy tradeoff

The choice of the SBR range on which to train SBR-Net was decided based on minimizing hallucination artifacts in the reconstructions of experimental data while also achieving good depth penetration. This is equivalent to balancing the robustness-accuracy tradeoff^39^, where, in our case, high robustness of the reconstruction model is measured by the level of hallucination artifacts and accuracy measured by imaging depth penetration and emitter 3D localization. Hallucination artifacts are a sign of poor robustness because the model falsely predicts emitters from background fluorescence. This model has not learned a general enough background feature space to reject background or a general enough target emitter feature space; instead, it is overly sensitive to minute background fluctuations and attributes them as emitters in a non-consistent manner. We experimented with three SBR ranges based on typical SBRs seen in scattering phantom experiments and *in vivo* rodent brain widefield imaging experiments. *In vivo* one-photon imaging experiments usually present neurons with very weak signal contrast, having SBRs between 1.05 and 2^5,7,16,26,31,40,41^, so we train one SBR-Net with a lower SBR range of 1.01 and 2.0. We also train an SBR-Net on a higher range between 2.0 – 3.0, and between 1.1 – 3.0, the latter being our main result.

**Fig. 6 |:**
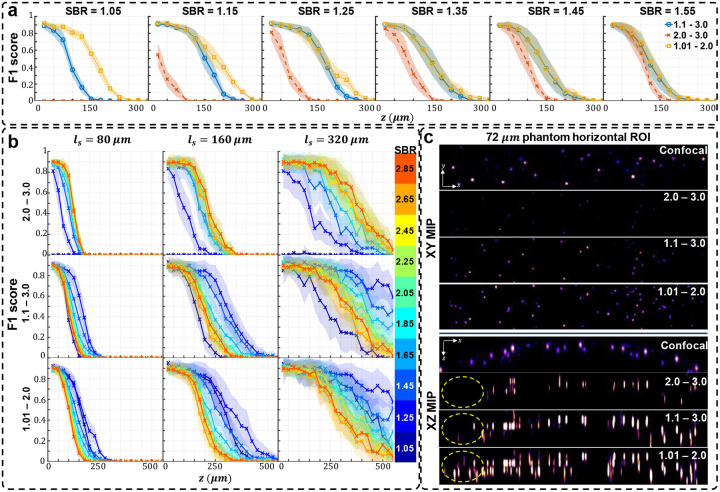
Analysis of networks trained on different SBR ranges. (a) The F1 score of each SBR-Net for across different SBR in simulation for scattering length of 80 μm. SBR-Net trained on the lowest range of 1.01 – 2.0 performs better than the other SBR-Nets. (b) On simulated testing data: F1 score of each SBR-Net (rows) for different scattering lengths (columns). SBR-Net (2.0 – 3.0) has a depth penetration performance that is intuitive, performing better on data with higher SBR. In contrast, SBR-Net (1.01 – 2.0) performs better on lower SBR than it does on higher SBR. SBR-Net (1.1 – 3.0) has better depth penetration for data with SBR of 1.25 and 1.35 compared to data with higher SBR; this is similar to the behavior of SBR-Net (1.01 – 2.0). However, for data with SBR 1.05 that is out of the training SBR range, it has the worse depth penetration performance compared to data with higher SBR. (c) On experimental testing data: XY and XZ MIPs of the reconstruction of the 72 μm scattering phantom. SBR-Net (2.0 – 3.0) behaves more conservatively, reconstructing emitters with high enough optical contrast and having fewer false positives at the expense of reconstructing lower SBR emitters. Examining the XY MIP of SBR-Net (1.01 – 2.0) reconstruction, it appears to reconstruct all the emitters that are there in the confocal measurement, in addition to more false positives, highlighted in the dashed yellow oval. The XZ MIP displays a clearer visualization of this hallucination behavior. Our main result is trained on SBR between 1.1 and 3.0, which is a balance between the aforementioned two cases in experimental data.

While the SBR-Net (1.01 – 2.0) performs better on synthetic data as shown in Fig. 6a–b, we observe many more false positives, or hallucinations when it is applied to experimental data, as seen in Fig. 6c. In other words, SBR-Net (1.01 – 2.0) has higher accuracy but lower robustness measure. In contrast, SBR-Net (2.0 – 3.0) when applied to experimental data performs more conservatively at the cost of depth penetration, having higher robustness at the cost of accuracy. In our main results, we balance this trade-off using an SBR-Net (1.1 – 3.0) trained on SBRs between 1.1 and 3.0. This network achieves depth penetration comparable to that in confocal microscopy with much fewer hallucination artifacts, as shown in Fig. 6c. An explanation for poor robustness of SBR-Net (1.01 – 2.0) is due to the model being sensitive to small deviations in the real measurement from the synthetic training data, such as refraction due to non-planar sample surface, optics and sample induced aberrations, unseen SBR measurement, and image sensor noise. Visually, the emitters are nearly imperceptible among background with SBR below 1.05. While this network performs well at depth penetration on synthetic test data that is generated from the same distribution as the training data, this sensitivity of emitter detection exacerbates the hallucination problem when the experimental measurement data has even small distributional differences from the synthetic training data. We further carry out reconstruction of the three SBR-Nets on the brain slice sample and observe the same robustness-accuracy tradeoff, shown in Extended Data Fig. 5.

We experimentally observe that the air-sample interfacial refraction is a significant source of experimental deviation from the synthetic training data, as our scattering model is built upon a free space model with the point spread functions (PSFs) measured in air. As a result, the lateral displacements of the emitters’ signals in the actual measurement do not follow with the axial shearing geometry of the 3D PSF, which leads to hallucinated emitters that are adjacent to the true positives, as evident in Fig. 6c of the XY MIP 2D reconstruction, which worsen towards the edge of the FOV and as the SBR range decreases. For the brain slice sample, which is flat, we do not observe such hallucination artifacts in the XY MIP 2D reconstruction as the SBR range decreases as shown in Extended Data Fig. 5 because there is minimal air-sample interfacial refraction.

### Network design factors affect bias-variance tradeoff

#### Number of unique training data pairs

It is well-known that the amount of training data has a significant impact on neural network generalization performance^42^. Therefore, we experiment with the number of unique training data pairs to test the model’s generalization performance. The goal is to balance this bias-variance tradeoff, where a good model has a high enough bias to produce low variance predictions, being more tolerant of out-of-distribution (OOD) experimental data. We find that 500 unique training data pairs allows good generalization to experimental data because there is not a diversity of features to learn, and we wish to avoid overfitting to synthetic training data as the neural network’s behavior will become too brittle to generalize to experimental data, which has small distributional differences with synthetic data.

We are mainly interested in learning features similar to neuronal bodies and background fluorescence, allowing the learning effort to require fewer examples compared to typical computer vision tasks that require large image datasets with more diverse features like Imagenet^43^, for example. We carry out an experiment where we change only the number of unique training data pairs; our main result is trained on 500 unique pairs (80/20, train/validate) and we train the same network with 1500 unique pairs (80/20, train/validate). We specify “unique” pairs because convolutional neural networks (CNNs) are equivariant in translation. This means that even though 224 × 224 pixel patches from the entire 512 × 512 data are chosen randomly online during training, allowing a larger number of different examples for the network to learn from, smaller sub-patches are seen multiple times in different locations over different epochs, which does not contribute to an overall lower validation loss due to the translation equivariance of our CNN with 3 × 3 convolution kernels.

**Extended Data Fig. 6 |:**
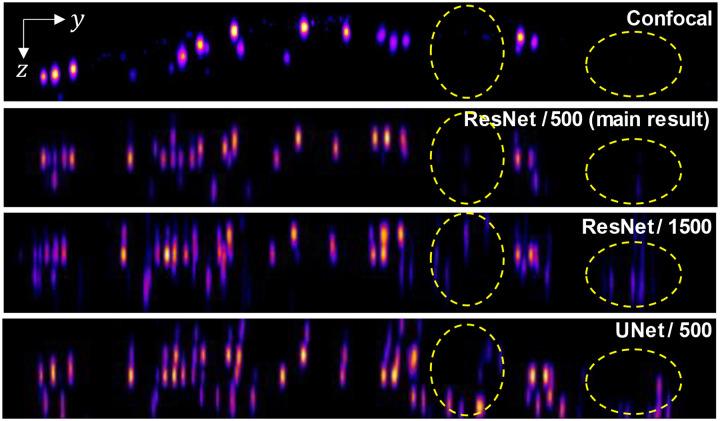
YZ MIPs of the SBR-Net reconstruction results on the 72 μm phantom SBR-Net trained on 1500 unique training data pairs generalizes more poorly to experimental data compared to the one trained with 500 unique pairs, as seen by more hallucination artifacts. We also see a similar behavior for the UNet-based SBR-Net with more network parameters.

Extended Data Fig. 6 shows that the SBR-Net trained on 1500 unique pairs suffers from hallucination artifacts, demonstrating high variance predictions and poor generalization to experimental data compared to our main result of an SBR-Net trained on only 500 unique pairs. This is an indication that the bias of the model trained on 1500 pairs is lower, resulting in a less robust performance. From our analysis in the previous section, we understand that a lack of robustness results in lower precision scores, which is equivalent to more hallucination artifacts.

#### Network architecture: ResNet vs UNet

We also test how the network architecture affects the generalization performance by comparing our ResNet-based SBR-Net with one that is UNet-based. We find that while the UNet architecture performs better on synthetic test data in terms of depth penetration, as seen in Fig. S7, it generates many more hallucination artifacts for experimental data. One likely reason for the hallucinations is that this UNet-based model may also overfit to synthetic data due to having 34,558,008 parameters, which is 20x more than our ResNet-based architecture (1,732,872), providing the model with a very low bias, a similar rationale as before. While the UNet-based architecture may be more suitable to address the receptive field problem that we observed in our phantom experiments, its poor generalization to experimental data due to the sensitivity to OOD input data would require extensive tuning of several parameters to fit a specific experimental condition.

We can measure the level of overfitting to synthetic data by comparing the validation losses between the networks. In Fig. S8, we verify that the SBR-Net trained on 1500 data pairs and the UNet-based SBR-Net have lower validation losses, i.e., fitting to synthetic data more. While this is desirable in most deep learning tasks, this validation set is also *synthetic* data. On the other hand, we desire learning the shared features between synthetic and experimental data without the network having a low enough bias where it specializes in synthetic data features, resulting in high variance inferences on experimental data. If more training pairs were used to train a model or if the model had more parameters, we may still control the bias-variance tradeoff by early stopping or other forms of regularization. However, our validation set can only be synthetic data, so this strategy is not practical due to the lack of ground truth for experimental data to decide when to stop early.

We focus on experiments with overfitting rather than underfitting because in practice, underfitting is unlikely to occur due to the lack of diversity of features in our learning task. While our choice of network architecture and number of unique training pairs may not be optimal, these results demonstrate the necessary considerations one should take for a model trained on synthetic data to be robust to experimental data.

### Input measurement analysis

**Extended Data Fig. 7 |:**
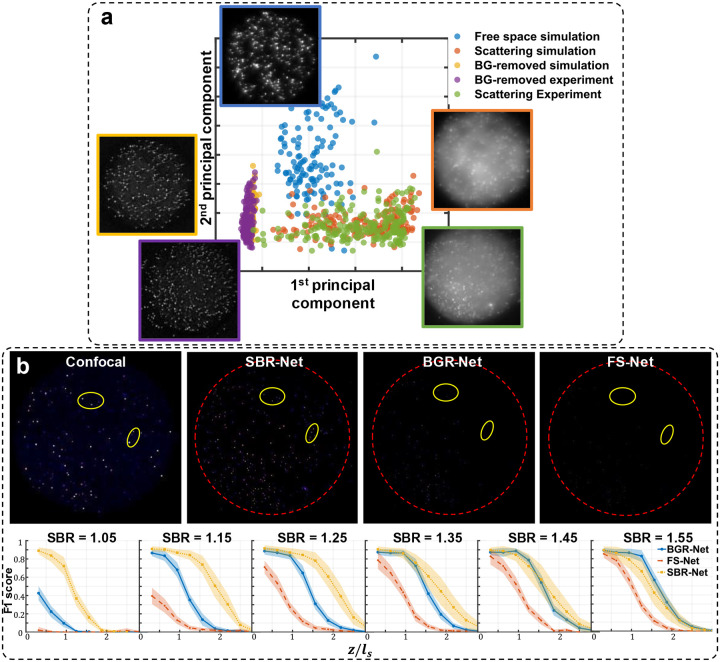
Dimensional reduction and BGR-Net reconstruction comparison. a) Principal component analysis of all measurement domains. b) Results of BGR-Net on the 72 μm phantom. Shown are the XY MIP reconstructions and F1 scores of BGR-Net compared to SBR-Net and FS-Net.

To gain a deeper understanding of the generalizability of our deep learning models, we perform dimensionality reduction through principal component analysis (PCA) for all measurements domains to analyze their similarities in an interpretable subspace. We compute the center location of all emitters in each measurement and retrieve a 32 × 32 pixel patch centered around it. Plotting each emitter’s first and second principal component, we see similar clustering for the synthetic and experimental scattering measurements as shown in Extended Data Fig. 7a, providing another explanation for how our model trained on synthetic data generalizes to real experimental data.

One thing we observe is that the synthetic free space data cluster is disjoint from the background removed data clusters. Background removed data is clearly an OOD input to FS-Net that was trained on synthetic free space data, and our qualitative observations of false positives from FS-Net reconstructions of synthetic data in Fig. 2 is consistent with experimental data where OOD inputs may lead to reconstructions with hallucinations.

Interestingly, we observe even more similar and tighter clustering between background-removed synthetic and experimental data. Naturally, we would want to explore if a network trained on scattering measurements that have undergone background removal would perform as well as SBR-Net. We term this network BGR-Net, for background-removed network, and visualize its XY MIP reconstructions of the experimental 72 μm phantom, using the confocal measurement as a reference baseline in Extended Data Fig. 7b. In simulation and experiment, BGR-Net is able to retain more emitters compared to FS-Net, but SBR-Net still performs better for reconstruction and localization. From the quantitative analysis of simulated data in Extended Data Fig. 7b, SBR-Net performs better than BGR-Net until a high enough SBR of around 1.55, where target emitters have more optical contrast and are less likely to be removed in the background removal step. However, for practical applications such as *in vivo* neural imaging, many neurons exhibit measurement SBRs lower than 1.55, rendering the proposed SBR-Net a more suitable approach for such a task.

## Discussion

We demonstrate a deep-learning-based single-shot 3D reconstruction algorithm, SBR-Net, to recover emitters embedded in scattering media with different scattering lengths. The SBR-Net is based on learning from synthetic data that was generated with our scattering simulator that captures the heterogenous low-contrast signal and strong background behavior of fluorescence measurements in imaging through tissue scattering applications. The experimentally achieved depth penetration and optical sectioning performance of the simulator-trained SBR-Net is similar to that of confocal microscopy, while requiring only a single measurement to reconstruct the 3D volume. Results on the brain slice experiment show favorable progress towards neuronal imaging applications in rodent brains, where target signals have very low contrast.

We analyze the generalization tradeoffs that arise from the choice of training data SBR range to discover that a larger range of SBRs between 1.1 and 3.0 lead to a more robust reconstruction for experimental data. Analyzing the effects of the number of training data pairs and number of model parameters reveals the well-known bias-variance tradeoff in machine learning. For our task of reconstructing spherical emitters and rejecting slowly varying background, we require fewer training data pairs and fewer model parameters since the diversity of learned features is limited and a more minimal learning structure is more suitable to generalize to experimental data and avoid overfitting to synthetic training data.

One caveat of our approach is that we only model the low-contrast behavior of scattered light. While this strategy has demonstrated favorable results in our experiments, we believe that for improved performance across a broader range of applications, it is necessary to model optical aberrations caused by the imaging optics and the sample^44^. Future work would investigate learning such aberrations based on physics-based simulator or experimental data, to incorporate them into a more robust model.

## Methods

### CM^2^ imaging system

We use our previously developed Computational Miniature Mesoscope (CM^2^) imaging system to demonstrate our descattering algorithm. Briefly, CM^2^ uses a 3×3 microlens array (MLA) as the sole imaging element to collect 9 tomographic views of the object volume, encoding 3D information in a single measurement. The numerical aperture (NA) of each lenslet is ~0.05, and the imaging system has a magnification of ~0.52. CM^2^ uses a Sony IMX-178 image sensor with a pixel size of 2.4 μm, rendering the object space sampling to be 4.15 μm, which satisfies the Nyquist sampling theorem for cellular level imaging targets between 8.5 and 25 μm. CM^2^ is equipped with an absorption and excitation filter that rejects light outside the emission bandwidth of green fluorescence and blue light, respectively. Further specifications and assembly instructions may be found on our open-source GitHub^45^.

Within a lateral field of view (FOV) of 2 mm in diameter, CM^2^’s system response is slice-wise shift-invariant^6^, so we constrain our objects of interest to be at most 2 mm in diameter. The magnification effect from the finite-conjugate nature of the MLA imaging system provides fast decorrelation along *z* which contributes to 3D resolution. Using a motorized *z*-stage and a 5 μm point source, we collect a defocused stack of on-axis point spread functions (PSF) from *z* = −225 μm to *z* = 375 μm with a 25 μm increment to characterize our system response in free space over an axial depth range of 600 μm. All measurements were taken with an exposure time of 50 ms and a digital gain of 1x.

### Forward model and training data generation

#### Forward model

Our scattering model is comprised of a free space, laterally shift-invariant forward model combined with added synthetic background to produce a scattering measurement *g* as

(1) $$g(u,v)=\alpha \sum_{z}PSF(u,v;z)*V(u,v;z)+BG$$

where * denotes a 2D discrete convolution, (*u*, *v*) are the pixel coordinates, *PSF*(*u*, *v;z*) is the measured free space PSF at the axial plane *z*, *V*(*u*, *v;z*) is the synthetic ground-truth volume of fluorescent emitters, *BG* is the synthetic background, and α is a scalar to control the SBR. The *BG* term is drawn from Gaussian-blurred random value noise (detailed below) where the blur kernel is uniformly distributed between 13 and 20 pixels (corresponding to 31.2 and 48 μm). The distribution of blur kernels accounts for background statistics observed in experimental phantoms across different bead densities and scattering conditions. The last step to generate the *BG* term is multiplying the Gaussian blurred result with a circular Gaussian envelope mask to account for the circular FOV of the object and the apodization of the imaging optics. Each measurement contains 2076 × 3088 pixels across an 8.6 × 12.8 mm object FOV.

We note that the NA of each lenslet in the MLA of CM^2^ is 0.05, which is low enough that we may neglect scattering-induced width broadening of the PSF^11^. For scattering-induced width-broadening to become significant enough in our imaging system, the depth range would have to be around 5 scattering lengths, which is beyond our application with one-photon imaging.

#### Value noise

For modeling background signals, we use value noise, which is a computer graphics tool to generate procedural textures by interpolating between integers on a random lattice^36^. Value noise is similar to Perlin noise, which has been used for modeling background fluorescence^46^. We use open source code^47^ to generate raw value noise of size 600 × 600 pixels, which corresponds to a measurement FOV of 1.4 mm × 1.4 mm. We show a sample of raw value noise and its low-pass filtered version in Fig. S1.

#### Synthetic training and testing data generation

All data synthesis and analysis were carried out in MATLAB R2019b. We generate *V*(*u*, *v;z*) by simulating spheres with diameters normally distributed around a mean of 15 μm with standard deviation (std) of 2 μm, and brightness normally distributed around a mean of 0.8 with std of 0.1. The spheres were first generated on a 5x finer grid with voxel size equal to 0.83 μm × 0.83 μm × 5 μm, then 5×5×5 average binned to make the ground-truth volume have the same discretization as our reconstruction. We generate our synthetic volumes with emitter densities normally distributed with mean 180 emitters/mm^3^ and std of 118 emitters/mm^3^.

To control the SBR of each synthetic measurement, we first normalize the free space measurement term (first term without *α*) and the background term in Eq. (1) to be between 0 and 1 before combining them. We compute the peak intensities of all emitters in the free space measurement using the MATLAB function imregionalmax and average the values to get the average target signal *S*. We compute the mean background signal *BG*_*mean*_ by averaging all the pixel values in the value noise sample. Defining SBR as

(2) $$SBR=\frac{\alpha S+BG_{\text{mean}}}{BG_{\text{mean}}},$$

we then calculate the value of *α* for a desired SBR of a scattering measurement to use in Eq. (1).

The final scattering measurement, *f*, includes signal-dependent Poisson-Gaussian noise^48^ which was added online during model training with noise parameters calibrated from experimental data using the following equation

(3) f=g+σ(g)ξ,

where *ξ* is independent noise drawn from a standard normal distribution, and *σ*(*g*) is the signal-dependent std given by

(4) $$\sigma (g)=\sqrt{ag+b},$$

where *a* and *b* are related to Poisson and Gaussian noise, respectively. We consider the *BG* term as signal for computing signal-dependent noise. Calibration of experimental measurements determined these parameters to be *a* = 1.49×10^−4^ ± 0.57×10^−4^ and *b* = 5.41×10^−6^ ± 2.78×10^−6^. This noise model accounts for comprehensive noise statistics present in digital imaging sensors. Poisson-Gaussian noise was added during network training in an online fashion where parameters *a* and *b* are randomized, and the measured uncertainties are the std for a normal distribution.

To generate free space synthetic training data, we use Eq. (1) with *BG* = 0 and α = 1, and the same steps are taken for adding noise.

For generating training data for BGR-Net, we first add Poisson-Gaussian noise to the scattering measurement offline, and then perform background removal as described later. No further noise was added online during training.

To generate the testing data, we add an additional signal attenuation model based on Beer-Lambert’s law, neglecting absorption, given by I(*z*) = I_0_/exp(−*z/l*_*s*_) where *l*_*s*_ is the scattering mean free path whose value is the length of the path of a ballistic photon where its intensity decays to 1/e. As a note, we do not include this attenuation for the training data so that we may have better control over the SBR of simulated measurements.

### Scattering phantom fabrication

We fabricate scattering phantoms with bulk scattering and background fluorescence. We control the bulk scattering using a combination of nonfluorescent and green fluorescent 1.1 μm polystyrene miscrospheres (i.e. scatterers) (Thermo Fisher Scientific, 5000 Series Polymer Particle Suspension; refractive index, 1.5979), and the background fluorescence with only the green fluorescent 1.1 μm microspheres. Our imaging target is a mixture of 10 and 15 μm green fluorescent polystyrene beads (Thermo Fisher Scientific, Fluoro-Max Dry Fluorescent Particles, 10 and 15 μm), and we neglect their contribution to bulk scattering.

We begin by centrifuging the nonfluorescent scatterer suspension (concentration 10% v/v) in a 1.5 mL microcentrifuge tube at 7000 rpm to separate the polystyrene microspheres from the water, and then pipette out the supernatant leaving only the solid scatterers. We then add 20 μL of the fluorescent scatterer suspension (concentration 1% solid), and the imaging targets. Finally, we add 1 mL of uncured polydimethylsiloxane (PDMS) with a 10:1 ratio of base to curing agent (Sylgard 184, Dow Corning Corp., Midland, MI, refractive index, 1.43). We use an ultrasonic probe sonicator (Fisherbrand^™^ Model 50 Sonic Dismembrator) to thoroughly mix the solution. This mixing process causes a significant amount of cavitation which makes the solution more scattering than desired, so we leave the solution in a vacuum degassing chamber for 20 minutes at a pressure of −30 bar to remove microscopic bubbles and any remaining water. We finally place 2 μL of the uncured solution inside a custom 3D printed ring of diameter 2 mm and height 0.5 mm and leave it in an oven at 40C for 4 hours to cure.

To calculate the amount of scatterers needed for a certain bulk scattering length, *l*_*s*_, we use Mie theory^49^:

(5) $$\phi =\frac{2d}{3l_{s}Q}$$

where *ϕ* is the concentration (v/v) of scatterers in the medium, d=1.1 μm is the diameter of a scatterer, and Q=1.9191 is the scattering efficiency computed with the online Mie scattering calculator^50^.

Our target scattering lengths for the phantoms are 75, 200 and 325 μm, for which we add 51, 19 and 12 μL respectively, of non-fluorescent scatterer suspension to 1 mL of PDMS. After adding 20 μL of fluorescent scatterers to each solution, the final scattering length is calculated to be 72, 181, and 279 μm, all with an anisotropy factor of *g* = 0.95795. In Fig. S6, we show confocal measurements of all three phantoms to verify that the target scattering length is achieved.

### Ex-vivo fixed rodent brain slice preparation

Histological sections with GFP-expressing neurons in the bed nucleus of the stria terminalis (BNST) were prepared using viral-mediated techniques. Male C57Bl/6 mice were anesthetized under continuous 2% isoflurane vapor and given preoperative analgesia (buprenorphine and ketoprofen, 0.5 and 5 mg/kg respectively). The head was secured in a digital stereotaxic apparatus (David Kopf Instruments, Tujunga, CA, USA), the skull was exposed with a midline incision and a small craniotomy was performed bilaterally over each injection site. Viral solutions (100–200 nL, pAAV-CAG-GFP, Addgene # 37825-AAVrg) were delivered to the BNST using a pulled glass micropipette (20 m tip diameter) and a Nanoject II (Drummond) positioned at coordinates 1.0 mm lateral from the midline, 0.4 mm anterior to bregma, and a depth of 4.3 mm from dura. The retrograde virus labeled both local neurons at the injection site in BNST as well as upstream brain areas. Animals recovered for two to three weeks after surgery to allow for viral expression and were then deeply anesthetized with ketamine-xylazine (100 and 15 mg/kg, respectively) and perfused transcardially with 4% paraformaldehyde in phosphate-buffered saline and their brains were subsequently removed, fixed, cryoprotected and sectioned coronally at 75 μm thickness using a cryostat. Sections were mounted on Superfrost Plus slides (Fisher) and Vectashield mounting medium (Vector Labs).

This study was performed in strict accordance with the recommendations in the Guide for the Care and Use of Laboratory Animals of the National Institutes of Health. All animals were handled according to approved Institutional Animal Care and Use Committee (IACUC) protocols (#201800540) of Boston University.

### Image processing-based background removal

Background removal was carried out in three steps using MATLAB R2019b. The first step is to perform image erosion (imerode) on the raw measurement with a disk structural element with radius approximately the same as that of the target structure, which is 5 pixels. Step 2 is to perform image dilation (imdilate) on the result of step 1 using the same structural element, and the last step is to subtract the output of step 2 from the original raw image.

### Light field refocusing

Light field refocusing is a backprojection algorithm for light field measurement. We implement this using a “shift-and-add” algorithm by the following equation:

(6) $$RFV(x,y,\Delta z)=\sum_{-1}^{1}\sum_{-1}^{1}VS\left(u,v;x-\frac{M^{2}d}{z_{0}}u\Delta z,y-\frac{M^{2}d}{z_{0}}v\Delta z\right)$$

where *RFV*(*x*, *y*, Δ*z*) is the refocused slice with Δ*z* refocused distance, *z*_*0*_ is the nominal focal distance from the MLA, *d* is the diameter of a lenslet in the MLA, *M* is the magnification of the imaging system, *VS* is the stack of cropped tomographic views, and (*u*, *v*) is the coordinates of the 3 × 3 MLA (i.e. *VS*(−1, −1;·,·) is the 512×512 cropped top left view, *VS*(0, 0;·,·) is the cropped center view, etc).

### Network training and implementation

#### Network architecture

Our network design follows a 2D ResNet structure^6^ where the depth dimension is the channel dimension of the tensors. The network design has two branches that take as input different but equivalent forms of the measurement, and then fused together in a final layer. Each branch is comprised of 20 ResBlocks that contain, in order: a 3×3 convolutional layer, batch normalization (BN), ReLU, another 3×3 convolutional layer, and BN. The input to each ResBlock is added to the output of the same ResBlock. The inputs for the two branches are the stack of 9 views, and the light field refocused volume, whose channel dimension are expanded from 9 to 48, and 24 to 48, respectively, in an initial expansion 3×3 convolutional layer. The input to the first ResBlock is added to the output of the 20^th^ ResBlock. After 20 ResBlocks, the branches are fused together and a final 3×3 convolutional layer squeezes the 48 channels to 24, which are the number of axial slices in the ground-truth volume. An illustration of the architecture is shown in Fig. S4. There are in total N = 42 3×3 convolutional layers along the forward path of the network, resulting in a receptive field of 83 pixels ([kernel_size − 1] × N − 1), or 344.45 μm.

The loss function we use is cross-entropy, which has shown to promote sparsity in the reconstruction^20^, and is defined by

(7) $$CE(y,\hat{y})=\sum_{i}y_{i}\text{ log}{\hat{y}}_{\iota }+\left(1-y_{i}\right)\text{ log}\left(1-{\hat{y}}_{\iota }\right)$$

where *y*_*i*_ and ${\hat{y}}_{\iota }$ are the ground truth and reconstruction intensity, respectively, at voxel *i*.

#### Training details

Using the method described previously, we generate 500 pairs of paired scattering training data for training SBR-Net, and 500 pairs of free space paired training data for FS-Net. We also take the 500 scattering measurements and perform background removal to train BGR-Net. During training, the 500 pairs were split 80/20 for training and validation. All three networks have the same architecture and training parameters. Additive Poisson-Gaussian noise was added online during training for SBR-Net and FS-Net as described previously. We also demonstrate a variance stabilization trick to substantially improve ResNet-based training loss convergence, shown in Fig. S9, which we discuss in detail in the supplementary information.

Training was carried out on a single NVIDIA V100 GPU with 16 GB memory using the PyTorch 1.9.0 framework. We initialize the weights using He initialization, which is optimized for ReLU non-linearities to prevent exploding or vanishing forward propagation signals that, in turn, affect the backpropagation signal^51^. We set the bias for each convolutional layer to be false. The inputs are B × C × 224 × 224 patches from the original B × C × 512 × 512 data, and the patch locations are randomized online during training. The batch size was heuristically set to be 12 to maximize memory availability on the GPU. The data and the weights of the network are single precision floating point numbers at 16-bit, which we accomplish using PyTorch’s automatic mixed precision (AMP) package. We use Adam for the optimizer and cosine annealing for the training schedule with an initial learning rate of 1×10^−3^ and a period of 30 epochs. The cosine annealing learning rate scheduler is able to more meaningfully explore the non-convex loss manifold over different local minima by providing the learning rate schedule with “warm restarts” that allows the model weights to escape local minima^52^. The validation loss was initialized to infinity and the model weights are saved every time the validation loss is lower than the current validation loss, so that the weights with the lowest local minimum is saved in case the network converges to a suboptimal local minimum. We set the maximum training time to be 48 hours and SBR-Net settled on a local minimum that was found after 28 hours, FS-Net 39 hours, and BGR-Net 12.5 hours. We also ran an experiment with a UNet-based architecture (see supplementary information) and the model weights were found after 44 hours.

During inference, the entire C × 512 × 512 data is passed through the network. For FS-Net and BGR-Net, we remove the background and then pass the input to the networks. For SBR-Net, we pass the raw, low-SBR scattering measurement input to the network. The inference runtime for one volume is 0.11 seconds on average using an Intel Xeon E5–1620 v4 3.5GHz CPU and 0.022 seconds on average using a Nvidia Quadro RTX 8000 48GB GPU. All MIP images of the reconstructions were visualized using Volume Viewer in ImageJ.

### Model-based reconstruction

We use the free space model for CM^2^ imaging system and invert the scattering measurements that have undergone background removal, both of which have been reported in our previous study^5^. Briefly, the imaging system is a slice-wise shift-invariant model, followed by a cropping operator, **C**, to account for the finite area of the camera sensor:

(8) y=CAx

where **x = [x**_1_**, x**_2_**, …, x**_n_**]** is the discretized 3D object with *n* number of axial slices and **A = [A**_1,_ **A**_2**, …,**_ **A**_n,_**]** is the slice-wise discrete forward model such that $Ax=\sum_{i=1}^{n}A_{\text{i}}x_{\text{i}}$, projecting all the depth-wise 2D measurements onto the camera sensor plane.

The inverse problem is highly ill-posed due to the 2D-to-3D dimension mismatch, so we incorporate sparsity priors in the spatial and gradient domain of the object and solve the following optimization problem:

(9) $$\hat{x}=\underset{x\geq 0}{\text{argmin}}\frac{1}{2}∥y-CAx{∥}_{2}^{2}+\tau_{1}∥x{∥}_{1}+\tau_{2}∥Dx{∥}_{1}$$

where **D** is the 3D finite difference operator, and *τ*_1_ and *τ*_2_ are manually tuned non-negative regularization parameters. We use a spatial sparsity prior because our objects are beads or neurons in a large volume, where there are many more zero-valued voxels than nonzero-valued. The 3D total variation prior is widely used for natural objects. We solve this optimization using our previously developed algorithm^5^ based on alternating direction method of multipliers (ADMM) which takes approximately 1.45 hr on the same Intel CPU we use for our DL-based reconstruction. More details are in the supplementary information.

### Detection metrics

The F1 score is our measure of performance for our reconstruction algorithms, which we perform on synthetic test data. F1 score is defined as

(10) $$\text{F}1\text{ score}=2\cdot \frac{\text{precision }\cdot \text{ recall}}{\text{precision}+\text{recall}}$$

where precision is defined as # true positives / (# true positives + # false positives) and recall is defined as # true positives / (# true positives + # false negatives).

We compute precision and recall slice-wise as well as for the entire volume by solving the linear assignment problem using MATLAB’s built-in matchpairs function. The 3D locations of the emitters are known for the ground truth, and the locations for the emitters in the reconstruction are computed by using the MATLAB function bwconncomp on thresholded binary reconstruction and then the emitter locations are given using the MATLAB function regionprops. Emitters that have fewer than 3 pixels are discarded to account for noise in the reconstruction. We carry out F1 score calculations using five threshold values, (0.1, 0.3, 0.5, 0.7, 0.9) and we use the result with the highest F1 score.

### Confocal microscopy

The Olympus FV3000 laser scanning confocal microscope was used to measure all scattering phantoms and the fixed brain slice. We used a 10x objective with numerical aperture of 0.4. The laser had a wavelength of 488nm operating at a power of 1.94 mW for all depths. The detector high voltage (HV) was set to 350 V, an offset of 4 V, and digital gain 1x. The final measurement was performed by collecting 2×2 tiles with a 100 μm overlap. Each tile was acquired using a 50 μm pinhole with a dwell time of 2 μs, a pixel sampling of 512×512 over a 1279 μm × 1279 μm FOV and an axial step size of 3 μm. All three phantoms and the fixed brain slice were measured with the same parameters except the number of axial slices, which were 51, 96, and 146, in order of increasing scattering length, and 30 for the brain slice.

## Extended Data

## Supplementary Material

1

## Figures and Tables

**Fig. 1 |:**
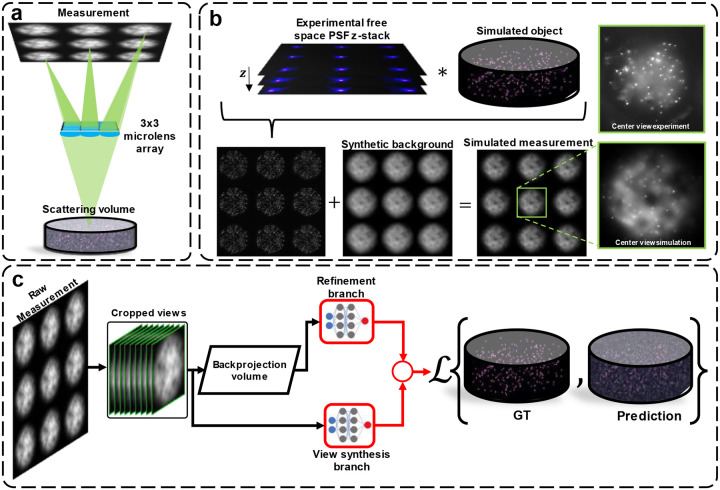
Overview of our forward and inverse model. (a) The lightfield imaging geometry of CM^2^ captures 3×3 views in each 2D measurement by a microlens array. (b) The imaging forward model for generating synthetic data. The final synthetic scattering measurement is the sum of synthetic free space measurement and synthetic background. A visualization comparing simulated and experimental data is also shown. (c) Our SBR-Net reconstruction pipeline fuses light-field refocusing enhancement and parallax information from the 3×3 views to reconstruct objects from low-SBR measurements.
