# Supplementary material for: Robust single-shot 3D fluorescence imaging in scattering media with a simulator-trained neural network
Source: ArXiv. 2023 Mar 22:arXiv:2303.12573v1. Preprint. [Version 1] (PMC10055497)
Supplement: Supplementary file 1 [file NIHPP2303.12573V1-supplement-1.pdf]

Supplementary Information: Robust 3D fluorescent imaging through scattering with deep learning on synthetic training data

Jeffrey Alido[1], Joseph Greene[1], Yujia Xue[1], Guorong Hu[1], Yunzhe Li[1], Kevin J. Monk[2], Brett T. DeBenedicts[2], Ian G. Davison[2,3], and Lei Tian[1,3]

[1]Department of Electrical and Computer Engineering, Boston University, Boston, MA 02215, USA.
[2]Department of Biology, Boston University, Boston, MA 02215, USA.
[3]Department of Biomedical Engineering, Boston University, Boston, MA 02215, USA.
[*]Correspondence: leitian@bu.edu, Tel.: 1-617-353-1334

March 23, 2023

Contents

S1 Value noise visualization **39**

S2 Statistical comparison of synthetic and experimental data **40**

S3 Network architecture **42**

S4 Synthetic test data precision and recall **43**

S5 Confocal measurement of experimental scattering phantoms **44**

S6 Effect of deep learning factors on SBR-Net generalization to experimental data **45**

S7 Variance stabilization speeds up training convergence **46**

S8 Model-based reconstruction **48**

S1 Value noise visualization

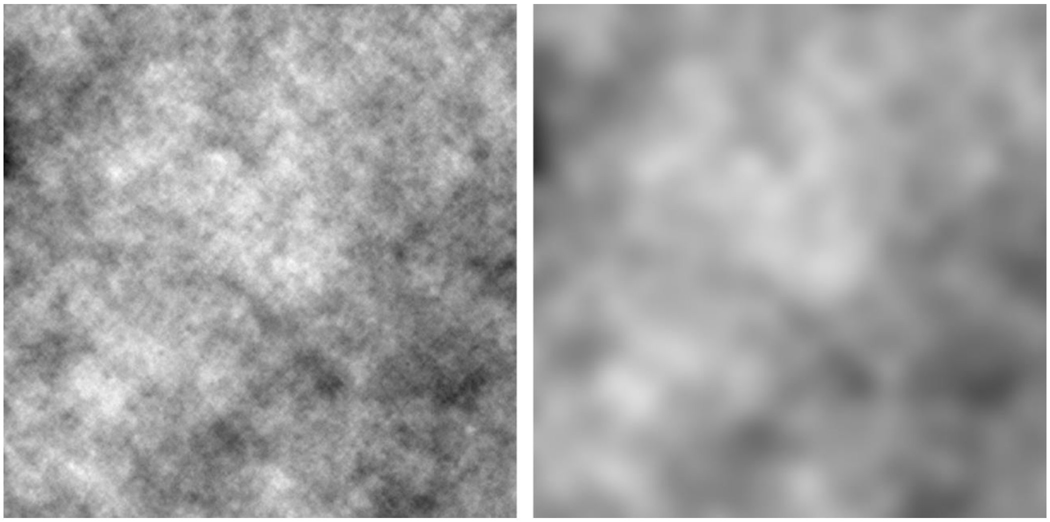

Figure S1: Raw 600 x 600 value noise and its low-pass filtered result using a Gaussian kernel with std of 13 pixels. Note the spatially varying intensities which contributes to different local SBRs.

S2 Statistical comparison of synthetic and experimental data

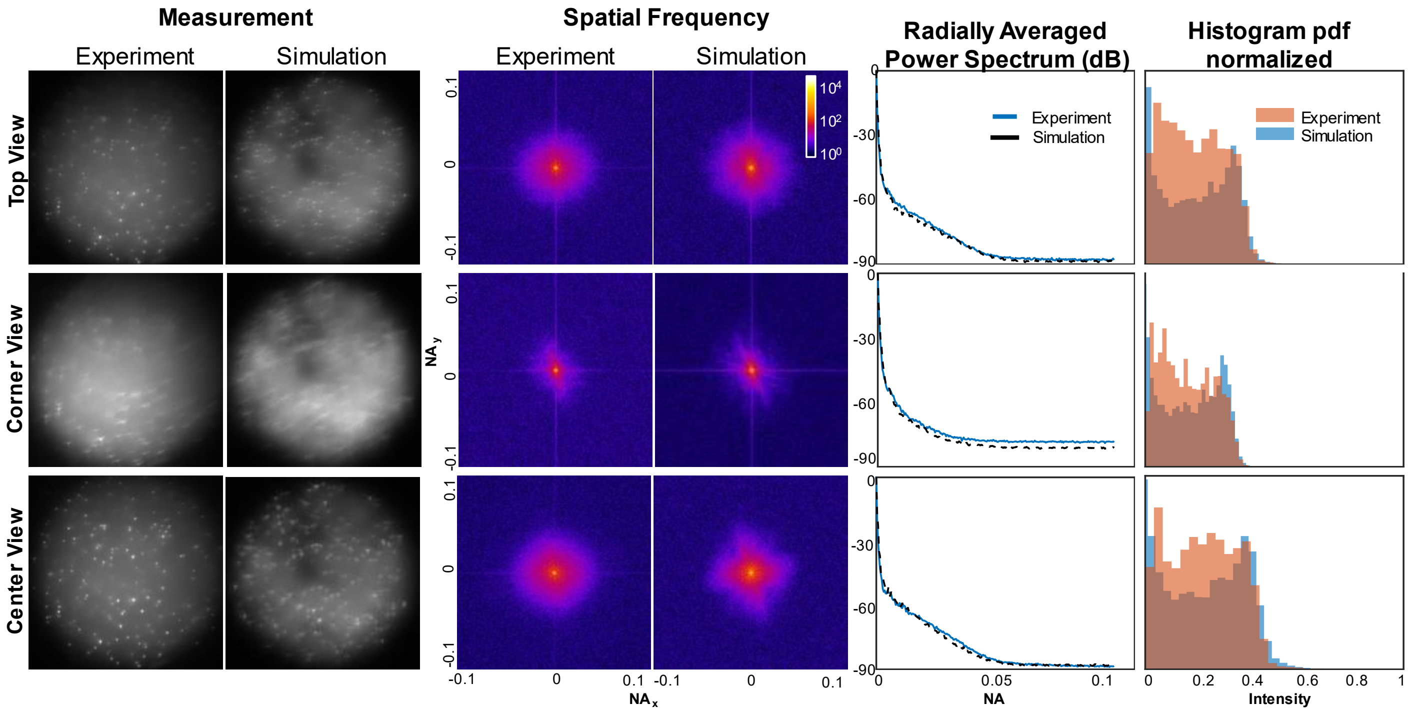

Figure S2: Qualitative and quantitative comparison of synthetic and experimental data for $l_s = 182\,\mu\text{m}$. We show the spatial frequency, power spectra, and intensity histograms for different views of a CM^2 measurement and observe strong similarities.

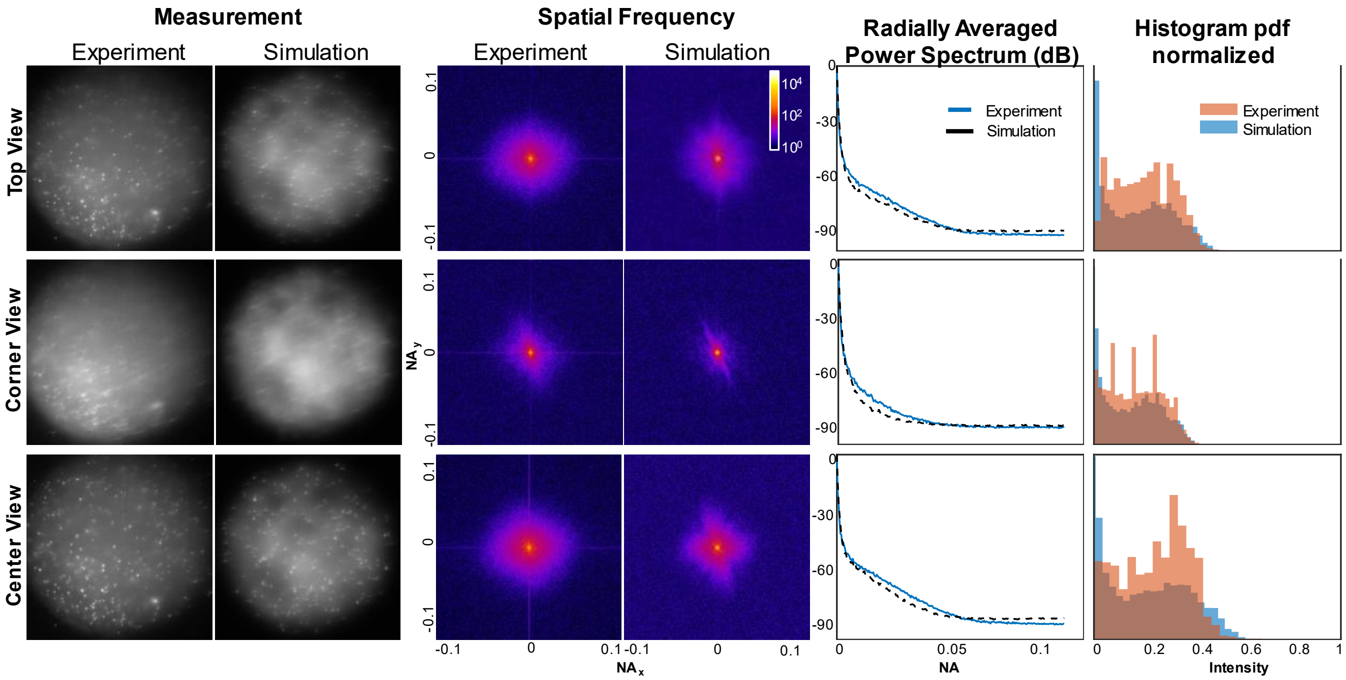

Figure S3: Qualitative and quantitative comparison of synthetic and experimental data for $l_s = 72\,\mu\text{m}$. We show the spatial frequency, power spectra, and intensity histograms for different views of a CM^2 measurement and observe strong similarities.

S3 Network architecture

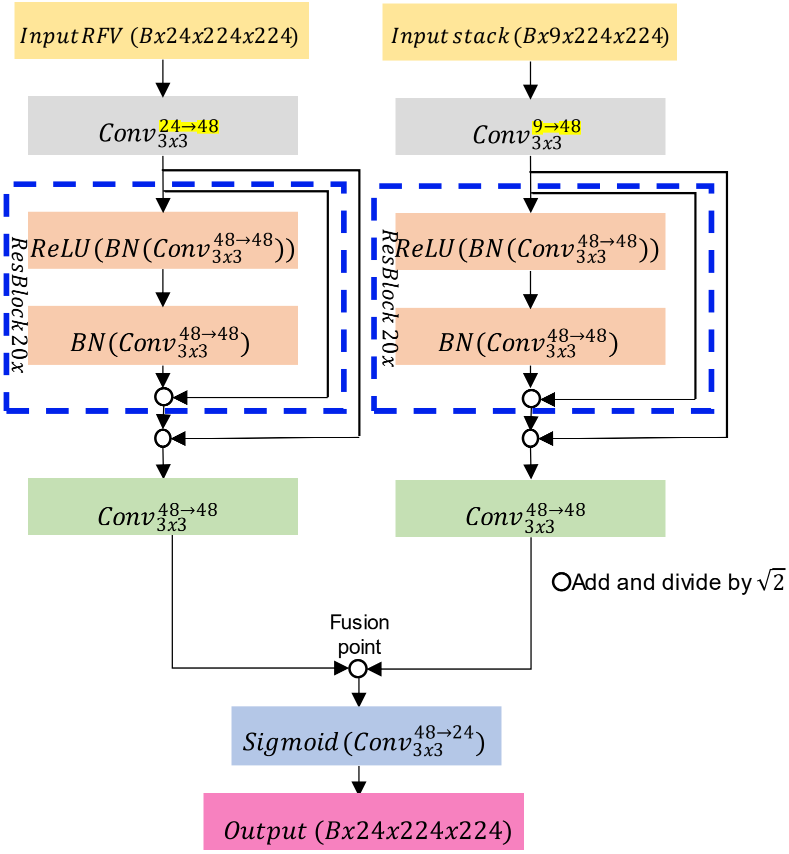

Figure S4: Schematic of our CNN architecture. The superscript of the convolutional layers denotes the number of input channels to the number of output channels, and the subscript denotes the kernel size. BN denotes a batch normalization layer, and B denotes batch size number.

S4 Synthetic test data precision and recall

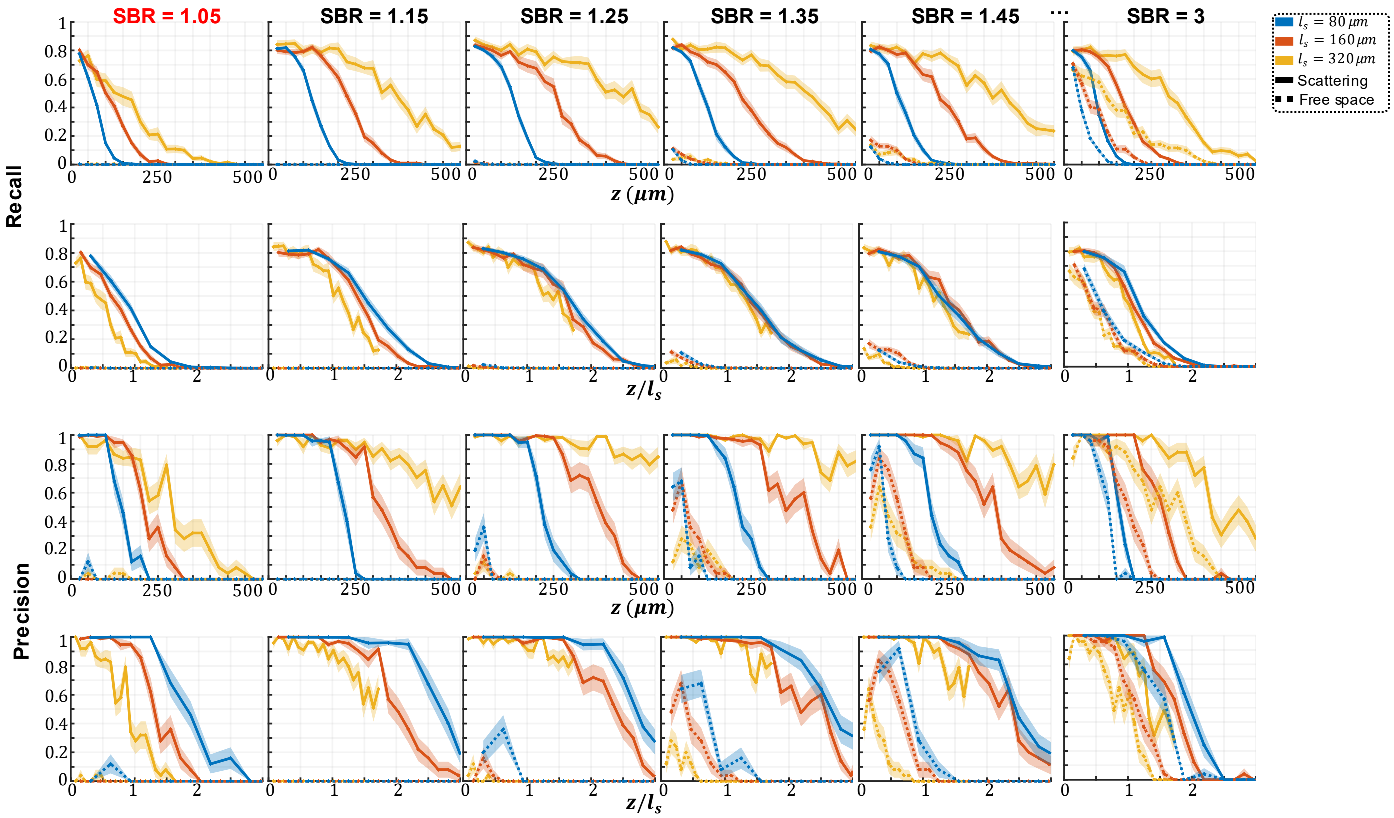

Figure S5: Simulation test data recall and precision metrics. The labeled SBR values are the average SBR values for the particles in the first depth layer before any optical attenuation. Similar to the optical signal, the SBR of a particle would decay exponentially over increasing depth of the particle, which is why we observe a systematic decay of performance for all scattering lengths, as seen in the normalized depths curves. Shaded regions represent standard error over 25 statistical samples. Note that the precision curve for free space peaks at a consistent depth, indicating the network's preference over objects with that depth's system response.

S5 Confocal measurement of experimental scattering phantoms

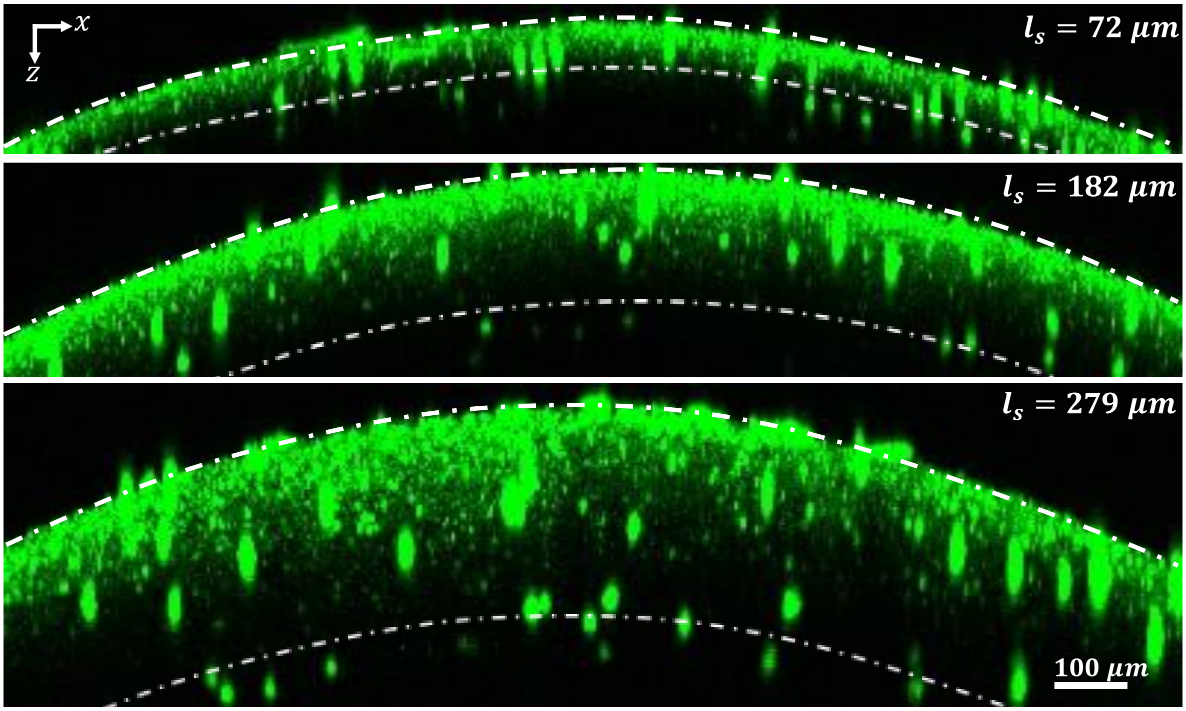

Figure S6: XZ MIP of 1600 µm × 50 µm ROI of the confocal measurements of the scattering phantoms. The dashed-dotted shell represents 1 scattering length from the surface of the phantom. We observe reasonable intensity attenuation that follows Beer-Lambert's law.

S6 Effect of deep learning factors on SBR-Net generalization to experimental data

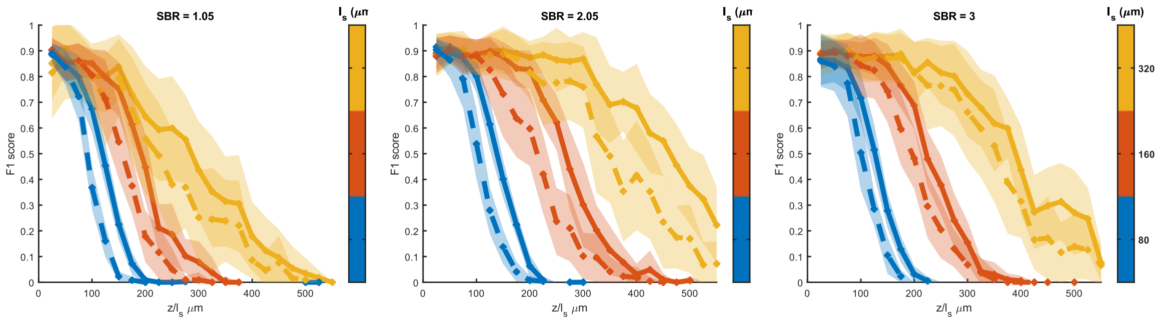

Figure S7: F1 scores for UNet-based (solid) and ResNet-based (dashed) architectures for data with different peak SBRs (1.05, 2.05, 3.0) across 3 scattering lengths (320, 160, 80 µm).

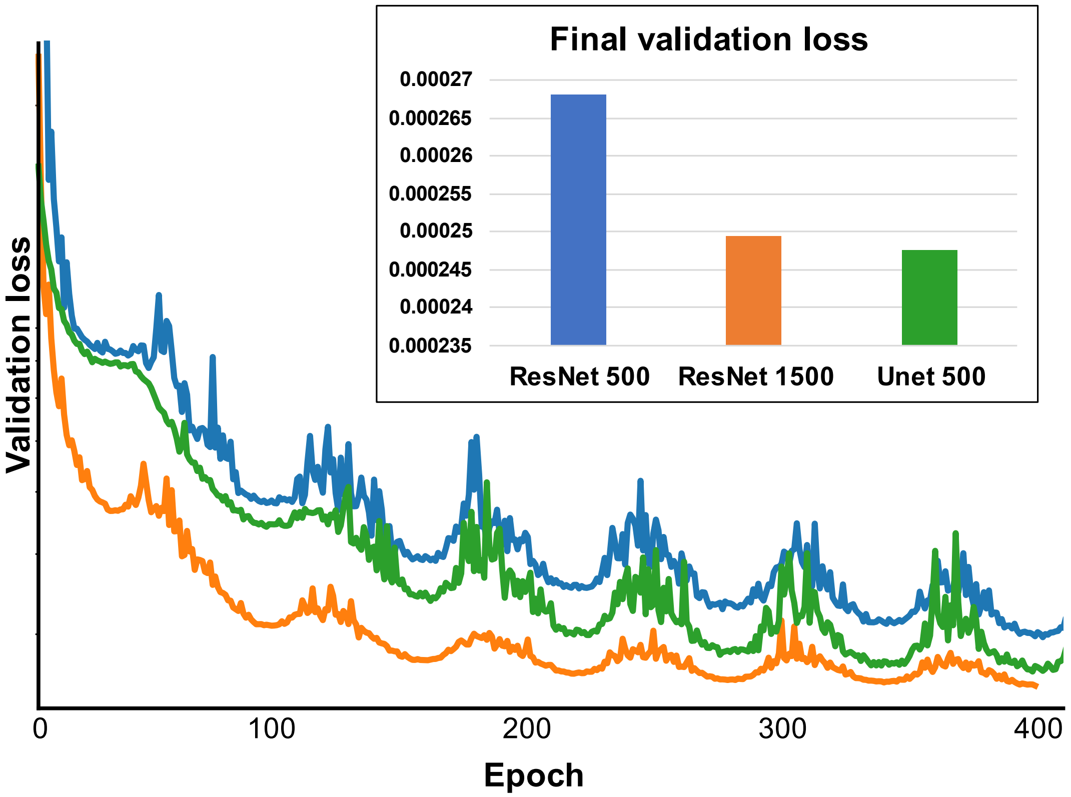

Figure S8: Validation loss for SBR-Net (500 pairs), SBR-Net (1500 pairs), and UNet-based SBR-Net.

S7 Variance stabilization speeds up training convergence

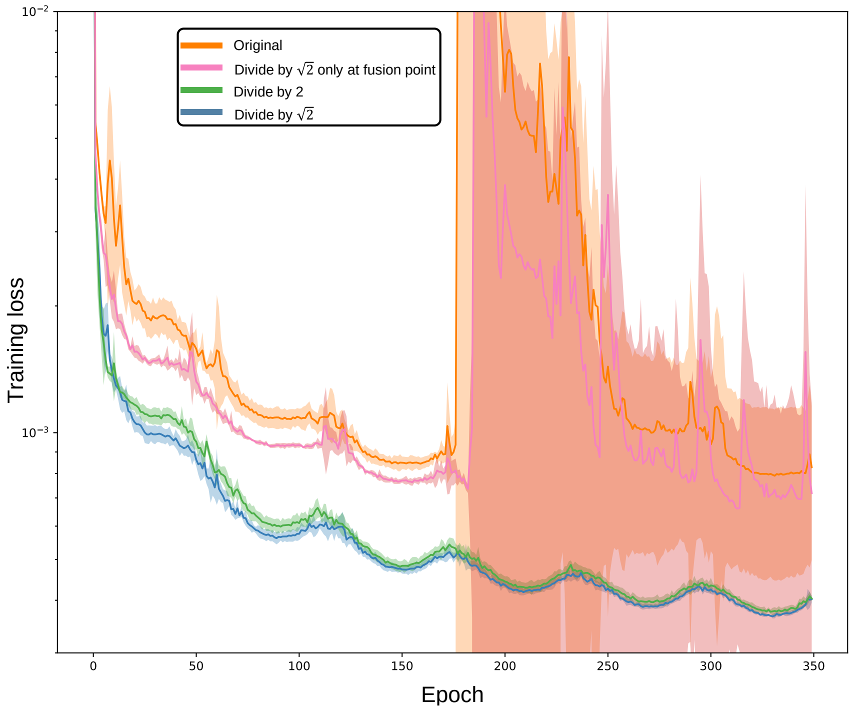

Figure S9: Variance stabilization speeds up training convergence by a factor of 7. Shading represents standard deviation for 5 training experiments.

When two independent random variables, X and Y are added, the variance of the sum, Z is

$$\begin{aligned} Var[Z] &= Var[aX] + Var[bY] \\ &= a^2 Var[X] + b^2 Var[Y]. \end{aligned} \tag{1}$$

In our ResNet-based CNN architecture, there are many channels with random elements being added, and we can safely assume they are independent during the first few epochs. When this happens, the variance grows rapidly over the 20 ResBlocks as well as with the branch fusion. The consequence is that the forward propagation signal has a significantly large range of values which may saturate and flood the sigmoid function at the end of the network, resulting in less meaningful backpropagation signals that lead to slower convergence to local minima.

In our case, a and b are equal to 1, and X and Y have the same variance, σ^2, so the variance of the forward propagation signal doubles after every ResBlock. We initialize the weights according to Kaiming He initialization [1], $W \sim \mathcal{N}(0, \frac{2}{n_l})$, where W is an element of the CNN layer and n_l is the number of elements in that layer. By the end, the output magnifies the input variance by 2^{21} (20 residual connections and one branch fusion). To ensure that the variance of the forward propagation signal does not explode and that we can take advantage of He initialization to its fullest in a ResNet-type architecture, we must ensure that the variance of the forward propagation signal is stabilized, meaning that the variance of the sum of two channels in the network architecture is the same as the variance of the channels that are being summed. From Equation 1, we see the solution is to divide the sum by $\sqrt{2}$:

$$Var[Z] = Var\left[\frac{X+Y}{\sqrt{2}}\right] = \frac{Var[X] + Var[Y]}{\sqrt{2}^2} = \frac{\sigma^2 + \sigma^2}{2} = \sigma^2. \tag{2}$$

Thus, for every part of our network where two channels are being added (i.e. the residual connections and the branch fusion), we simply divide the sum by $\sqrt{2}$. In general, channels being fused together with addition should have the sum divided by the square root of the number of channels being fused together.

We experiment with 4 different network structures: without variance stabilization, with variance stabilization for every residual connection and the branch fusion, variance stabilization at only the branch fusion point with the original ResNet residual connections, and dividing by 2 for all residual connections and the branch fusion. Figure S9 show the convergence of the training loss over epochs, and we see that the architecture with variance stabilization is optimal. It takes the architecture with variance stabilization 7 times faster to reach the same training loss as it does for the original architecture without variance stabilization. The original architecture and the one where only the branch fusion point has been divided by $\sqrt{2}$ are prone to severely non-optimal model weights due to the large variance of the forward propagation signal combined with warm restarts of the learning rate.

S8 Model-based reconstruction

This section adopts the following notation: bolded uppercase letters denote operators, a superscript of H denotes the Hermitian adjoint of the operator, bolded lowercase letters denote vectors, $\top$ denotes the transpose, and unbolded characters denote scalars.

The imaging system is a slice-wise shift-invariant model, followed by a cropping operator, $\mathbf{C}$, to account for the finite area of the camera sensor:

$$\mathbf{y} = \mathbf{CAx} \tag{3}$$

where $\mathbf{x} = [\mathbf{x}_1, \mathbf{x}_2, ..., \mathbf{x}_n]$ is the discretized 3D object with n number of axial slices, and $\mathbf{A} = [\mathbf{A}_1, \mathbf{A}_2, ..., \mathbf{A}_n]$ is the slice-wise discrete forward model such that $\mathbf{Ax} = \sum_{i=1}^{n} \mathbf{A}_i \mathbf{x}_i$, projecting all the depth-wise 2D measurements onto the camera sensor plane.

The inverse problem is highly ill-posed due to the 2D to 3D dimension mismatch, so we incorporate sparsity priors in the spatial and gradient domain of the object and solve the following optimization problem:

$$\hat{\mathbf{x}} = \underset{\mathbf{x} \geq 0}{\arg\min} \frac{1}{2} \|\mathbf{y} - \mathbf{CAx}\|_2^2 + \tau_1 \|\mathbf{x}\|_1 + \tau_2 \|\mathbf{Dx}\|_1 \tag{4}$$

where $\mathbf{D}$ is the 3D finite difference operator, and τ_1 and τ_2 are manually tuned non-negative regularization parameters. We use a spatial sparsity prior because our objects are beads or neurons in a volume, where there will be many more zero-valued voxels than nonzero-valued voxels. The 3D total variation prior is widely used for natural objects.

We solve this optimization problem using the alternating direction method of multipliers (ADMM) [2–4]. The first step is to perform variable splitting by setting $\mathbf{u} = \mathbf{Ax}$, $\mathbf{v} = \mathbf{x}$, $\mathbf{w} = \mathbf{Dx}$, $\mathbf{z} = \mathbf{x}$, and adding their corresponding constraint sets to the objective. We rewrite the optimization problem as

$$\begin{aligned} \underset{\mathbf{x}, \mathbf{u}, \mathbf{v}, \mathbf{w}, \mathbf{z}}{\arg\min} \quad & \frac{1}{2} \|\mathbf{y} - \mathbf{Cu}\|_2^2 + \tau_1 \|\mathbf{v}\|_1 + \tau_2 \|\mathbf{w}\|_1 \\ \text{s.t.} \quad & \mathbf{u} = \mathbf{Ax}, \\ & \mathbf{v} = \mathbf{x}, \\ & \mathbf{w} = \mathbf{Dx}, \\ & \mathbf{z} = \mathbf{x}, \\ & \mathbf{z} \geq 0. \end{aligned} \tag{5}$$

The next step is to form the augmented Lagrangian to make the optimization unconstrained:

$$\begin{aligned} \mathcal{L}(\{\mathbf{u}, \mathbf{v}, \mathbf{w}, \mathbf{z}, \mathbf{x}\}, \{\boldsymbol{\kappa}, \boldsymbol{\lambda}, \boldsymbol{\mu}, \boldsymbol{\nu}\}) = & \frac{1}{2} \|\mathbf{y} - \mathbf{Cu}\|_2^2 + \tau_1 \|\mathbf{v}\|_1 + \tau_2 \|\mathbf{w}\|_1 \\ & + \frac{\rho_1}{2} \|\mathbf{Ax} - \mathbf{u}\|_2^2 + \boldsymbol{\kappa}^\top (\mathbf{Ax} - \mathbf{u}) \\ & + \frac{\rho_2}{2} \|\mathbf{x} - \mathbf{v}\|_2^2 + \boldsymbol{\lambda}^\top (\mathbf{x} - \mathbf{v}) \\ & + \frac{\rho_3}{2} \|\mathbf{Dx} - \mathbf{w}\|_2^2 + \boldsymbol{\mu}^\top (\mathbf{Dx} - \mathbf{w}) \\ & + \frac{\rho_4}{2} \|\mathbf{x} - \mathbf{z}\|_2^2 + \boldsymbol{\nu}^\top (\mathbf{x} - \mathbf{z}) \\ & + \mathcal{I}_+(\mathbf{z}) \end{aligned} \tag{6}$$

where ρ_i are positive penalty parameters, $(\boldsymbol{\kappa}, \boldsymbol{\lambda}, \boldsymbol{\mu}, \boldsymbol{\nu})$ are the Lagrangian multipliers, or dual variables, and $\mathcal{I}_+(\mathbf{z})$ is the non-negativity barrier function defined as

$$\mathcal{I}_+(z) = \begin{cases} \infty & z < 0 \\ 0 & z \geq 0. \end{cases} \tag{7}$$

The dual function is defined as

$$g(\boldsymbol{\kappa}, \boldsymbol{\lambda}, \boldsymbol{\mu}, \boldsymbol{\nu}) = \inf_{\mathbf{u}, \mathbf{v}, \mathbf{w}, \mathbf{z}, \mathbf{x}} \mathcal{L}(\{\mathbf{u}, \mathbf{v}, \mathbf{w}, \mathbf{z}, \mathbf{x}\}, \{\boldsymbol{\kappa}, \boldsymbol{\lambda}, \boldsymbol{\mu}, \boldsymbol{\nu}\}) \tag{8}$$

and solving Eq. 4 is equivalent to solving the *dual problem*:

$$\max_{\boldsymbol{\kappa},\boldsymbol{\lambda},\boldsymbol{\mu},\boldsymbol{\nu}} g(\boldsymbol{\kappa},\boldsymbol{\lambda},\boldsymbol{\mu},\boldsymbol{\nu}). \tag{9}$$

The optimization problem is then a saddle point problem solved by iteratively alternating between a minimization step of the primal variables, followed by a maximization step of the dual variables,

$$\max_{\boldsymbol{\kappa},\boldsymbol{\lambda},\boldsymbol{\mu},\boldsymbol{\nu}} \min_{\mathbf{u},\mathbf{v},\mathbf{w},\mathbf{z},\mathbf{x}} \mathcal{L}(\{\mathbf{u},\mathbf{v},\mathbf{w},\mathbf{z},\mathbf{x}\},\{\boldsymbol{\kappa},\boldsymbol{\lambda},\boldsymbol{\mu},\boldsymbol{\nu}\}). \tag{10}$$

We update the dual variables using gradient ascent where the step size is the corresponding penalty parameter. Using calculus, we compute the update steps for the primal variables. The algorithm is as follows:

$$\begin{aligned}
\mathbf{u}_{k+1} &= \arg\min_{\mathbf{u}} \left\{ \frac{1}{2}\|\mathbf{y}-\mathbf{C}\mathbf{u}\|_2^2 + \frac{\rho_1}{2}\|\mathbf{A}\mathbf{x}_k-\mathbf{u}\|_2^2 + \boldsymbol{\kappa}_k^\top(\mathbf{A}\mathbf{x}_k-\mathbf{u}) \right\} \\
&= (\mathbf{C}^H\mathbf{C}+\rho_1\mathbf{I})^{-1}(\mathbf{C}^H\mathbf{y}_k + \boldsymbol{\kappa}_k + \rho_1\mathbf{A}\mathbf{x}_k) \\
\mathbf{v}_{k+1} &= \arg\min_{\mathbf{v}} \left\{ \tau_1\|\mathbf{v}\|_1 + \frac{\rho_2}{2}\|\mathbf{x}_k-\mathbf{v}\|_2^2 + \boldsymbol{\lambda}_k^\top(\mathbf{x}_k-\mathbf{v}) \right\} \\
&= \arg\min_{\mathbf{v}} \left\{ \frac{\tau_1}{\rho_2}\|\mathbf{v}\|_1 + \frac{1}{2}\|\mathbf{x}_k-\mathbf{v}+\frac{\boldsymbol{\lambda}_k}{\rho_2}\|_2^2 + \cancel{\frac{\boldsymbol{\lambda}_k^\top\boldsymbol{\lambda}_k}{\rho_2^2}} \right\} \\
&= \mathrm{Prox}_{\frac{\tau_1}{\rho_2}\|\cdot\|_1}\left(\mathbf{x}_k - \frac{\boldsymbol{\lambda}_k}{\rho_2}\right) \\
&= \mathcal{S}_{\frac{\tau_1}{\rho_2}}\left(\mathbf{x}_k - \frac{\boldsymbol{\lambda}_k}{\rho_2}\right) \\
\mathbf{w}_{k+1} &= \arg\min_{\mathbf{w}} \left\{ \tau_2\|\mathbf{w}\|_1 + \frac{\rho_3}{2}\|\mathbf{D}\mathbf{x}_k-\mathbf{w}\|_2^2 + \boldsymbol{\mu}_k^\top(\mathbf{D}\mathbf{x}_k-\mathbf{w}) \right\} \\
&= \arg\min_{\mathbf{w}} \left\{ \frac{\tau_2}{\rho_3}\|\mathbf{w}\|_1 + \frac{1}{2}\|\mathbf{D}\mathbf{x}_k-\mathbf{w}+\frac{\boldsymbol{\mu}_k}{\rho_3}\|_2^2 + \cancel{\frac{\boldsymbol{\mu}_k^\top\boldsymbol{\mu}_k}{\rho_3^2}} \right\} \\
&= \mathrm{Prox}_{\frac{\tau_2}{\rho_3}\|\cdot\|_1}\left(\mathbf{D}\mathbf{x}_k - \frac{\boldsymbol{\mu}_k}{\rho_3}\right) \\
&= \mathcal{S}_{\frac{\tau_2}{\rho_3}}\left(\mathbf{D}\mathbf{x}_k - \frac{\boldsymbol{\mu}_k}{\rho_3}\right) \\
\mathbf{z}_{k+1} &= \arg\min_{\mathbf{z}} \left\{ \frac{\rho_4}{2}\|\mathbf{x}_k-\mathbf{z}\|_2^2 + \boldsymbol{\nu}_k^\top(\mathbf{x}_k-\mathbf{z}) + \mathcal{I}_+(\mathbf{z}) \right\} \\
&= \arg\min_{\mathbf{z}} \left\{ \mathcal{I}_+(\mathbf{z}) + \|\mathbf{x}_k-\mathbf{z}+\frac{\boldsymbol{\nu}_k}{\rho_4}\|_2^2 + \cancel{\frac{\boldsymbol{\nu}_k^\top\boldsymbol{\nu}_k}{\rho_4}} \right\} \\
&= \max\left(\mathbf{0}, \mathbf{x}_k + \frac{\boldsymbol{\nu}_k}{\rho_4}\right) \\
\mathbf{x}_{k+1} &= \arg\min_{\mathbf{x}} \Big\{ \frac{\rho_1}{2}\|\mathbf{A}\mathbf{x}-\mathbf{u}_{k+1}\|_2^2 + \boldsymbol{\kappa}_{k+1}^\top(\mathbf{A}\mathbf{x}-\mathbf{u}_{k+1}) + \frac{\rho_2}{2}\|\mathbf{x}-\mathbf{v}_{k+1}\|_2^2 + \boldsymbol{\lambda}_{k+1}^\top(\mathbf{x}-\mathbf{v}_{k+1}) \\
&\qquad + \frac{\rho_3}{2}\|\mathbf{D}\mathbf{x}-\mathbf{w}_{k+1}\|_2^2 + \boldsymbol{\mu}_{k+1}^\top(\mathbf{D}\mathbf{x}-\mathbf{w}_{k+1}) + \frac{\rho_4}{2}\|\mathbf{x}-\mathbf{z}_{k+1}\|_2^2 + \boldsymbol{\nu}_{k+1}^\top(\mathbf{x}-\mathbf{z}_{k+1}) \Big\} \\
&= \frac{\mathbf{A}^H(\rho_1\mathbf{u}_{k+1}-\boldsymbol{\kappa}_k) + \mathbf{D}^H(\rho_3\mathbf{w}_{k+1}-\boldsymbol{\mu}_k) + \rho_2\mathbf{v}_{k+1} + \rho_4\mathbf{z}_{k+1} + \boldsymbol{\nu}_k - \boldsymbol{\lambda}_k}{\rho_1\mathbf{A}^H\mathbf{A} + \rho_2\mathbf{I} + \rho_3\mathbf{D}^H\mathbf{D} + \rho_4\mathbf{I}} \\
\boldsymbol{\kappa}_{k+1} &= \boldsymbol{\kappa}_k + \rho_1(\mathbf{A}\mathbf{x}_{k+1} - \mathbf{u}_{k+1}) \\
\boldsymbol{\lambda}_{k+1} &= \boldsymbol{\lambda}_k + \rho_2(\mathbf{x}_{k+1} - \mathbf{v}_{k+1}) \\
\boldsymbol{\mu}_{k+1} &= \boldsymbol{\mu}_k + \rho_3(\mathbf{D}\mathbf{x}_{k+1} - \mathbf{w}_{k+1}) \\
\boldsymbol{\nu}_{k+1} &= \boldsymbol{\nu}_k + \rho_4(\mathbf{x}_{k+1} - \mathbf{z}_{k+1})
\end{aligned} \tag{11}$$

where k is the iteration step and $\text{Prox}_{\alpha f}$ refers to the proximal operator of function αf. In our case, αf, is the $l1$ norm with regularization parameter, α. This is equal to the soft-thresholding function, $\mathcal{S}_\alpha$.

The adjoint of the cropping operator is zero-padding outside the sensor region, and $\mathbf{C}^H\mathbf{C}$ may be interpreted as a binary mask with a rectangle of ones in the center corresponding to the size of the sensor, so that the matrix is diagonalized and easily invertible. The finite difference operator, $\mathbf{D}$, is implemented using the forward difference operator (i.e. $x_{i+1} - x_i$), and its adjoint is the backwards difference operator, (i.e. $x_{i-1} - x_i$). We assume a circular boundary for simplicity. $\mathbf{D}^H\mathbf{D}$ may be implemented by using a convolutional kernel to carry out the $\mathbf{D}$ and $\mathbf{D}^H$ operator. The forward model operator, $\mathbf{A}$, the 3D finite difference operator $\mathbf{D}$, and their adjoints were carried out in the Fourier domain for computational efficiency and ease of invertibility when the matrix is diagonalized. Our implementation may be found in our open source GitHub [5].

We find the optimal regularization parameters through grid search and visually inspecting the most accurate reconstructions across all three scattering phantoms. The values are $\tau_1 = 0.01$, $\tau_2 = 0.005$, and we keep the penalty parameters $\rho_1 = \rho_2 = \rho_3 = \rho_4 = 1$. The algorithm is carried out in MATLAB R2019b using a Intel Xeon E5-1620 v4 3.5GHz CPU and takes approximately 1.45 hours for one volume.

References

[1] K. He, X. Zhang, S. Ren, and J. Sun, "Delving deep into rectifiers: Surpassing human-level performance on ImageNet classification."

[2] S. Boyd, "Distributed optimization and statistical learning via the alternating direction method of multipliers," vol. 3, no. 1, pp. 1–122.

[3] Y. Xue, I. G. Davison, D. A. Boas, and L. Tian, "Single-shot 3d wide-field fluorescence imaging with a computational miniature mesoscope," vol. 6, no. 43, p. eabb7508. Publisher: American Association for the Advancement of Science.

[4] N. Antipa, G. Kuo, R. Heckel, B. Mildenhall, E. Bostan, R. Ng, and L. Waller, "DiffuserCam: lensless single-exposure 3d imaging," vol. 5, no. 1, pp. 1–9. Publisher: Optica Publishing Group.

[5] "https://github.com/bu-cisl/computational-miniature-mesoscope-CM2."
